# Computational dissection of genetic variation modulating the response of multiple photosynthetic phenotypes to the light environment

**DOI:** 10.1186/s12864-024-09968-8

**Published:** 2024-01-20

**Authors:** Huiying Gong, Ziyang Zhou, Chenhao Bu, Deqiang Zhang, Qing Fang, Xiao-Yu Zhang, Yuepeng Song

**Affiliations:** 1 College of Science, Beijing Forestry University, No. 35, Qinghua East Road, Beijing, 100083 P. R. China; 2 College of Biological Sciences and Technology, Beijing Forestry University, No. 35, Qinghua East Road, Beijing, 100083 P. R. China; 3 Faculty of Science, Yamagata University, Yamagata, 990 Japan

**Keywords:** Photosynthesis, Electron transport rate, Photochemical quenching, Nonphotochemical quenching, Light environment, Genetic variation

## Abstract

**Background:**

The expression of biological traits is modulated by genetics as well as the environment, and the level of influence exerted by the latter may vary across characteristics. Photosynthetic traits in plants are complex quantitative traits that are regulated by both endogenous genetic factors and external environmental factors such as light intensity and CO_2_ concentration. The specific processes impacted occur dynamically and continuously as the growth of plants changes. Although studies have been conducted to explore the genetic regulatory mechanisms of individual photosynthetic traits or to evaluate the effects of certain environmental variables on photosynthetic traits, the systematic impact of environmental variables on the dynamic process of integrated plant growth and development has not been fully elucidated.

**Results:**

In this paper, we proposed a research framework to investigate the genetic mechanism of high-dimensional complex photosynthetic traits in response to the light environment at the genome level. We established a set of high-dimensional equations incorporating environmental regulators to integrate functional mapping and dynamic screening of gene‒environment complex systems to elucidate the process and pattern of intrinsic genetic regulatory mechanisms of three types of photosynthetic phenotypes of *Populus simonii* that varied with light intensity. Furthermore, a network structure was established to elucidate the crosstalk among significant QTLs that regulate photosynthetic phenotypic systems. Additionally, the detection of key QTLs governing the response of multiple phenotypes to the light environment, coupled with the intrinsic differences in genotype expression, provides valuable insights into the regulatory mechanisms that drive the transition of photosynthetic activity and photoprotection in the face of varying light intensity gradients.

**Conclusions:**

This paper offers a comprehensive approach to unraveling the genetic architecture of multidimensional variations in photosynthetic phenotypes, considering the combined impact of integrated environmental factors from multiple perspectives.

**Supplementary Information:**

The online version contains supplementary material available at 10.1186/s12864-024-09968-8.

## Background

Photosynthesis, as a crucial physiological process on Earth, serves as the fundamental basis for the sustenance of the biosphere. In recent years, research on photosynthesis has advanced significantly, leading to notable breakthroughs in the analysis of photosynthetic structure [1] and the elucidation of the genetic mechanisms underlying photosynthesis [2–5]. With the advancement of chlorophyll fluorescence technology, the investigation of photosynthesis has witnessed a new breakthrough through the analysis of photosynthetic phenotypes, including chlorophyll fluorescence parameters [6, 7]. Photosynthetic phenotypes represent intricate quantitative traits, wherein the quantitative characteristics of an organism are subject to a complex, multilevel, and multistage process involving intrinsic genetic control, reciprocal perturbations, and environmental influences. The complex and variable climate, along with diverse topography, gives rise to variations in environmental regulatory patterns, including temperature, humidity, altitude, and light intensity [8–10]. Capturing the dynamic processes of complex traits and constructing gene‒environmental regulation systems are of great significance for elucidating photosynthesis mechanisms and quantifying the patterns of change in high-dimensional, complex quantitative traits. This knowledge can provide valuable guidance for forest tree production and ecosystem management.

Genetic variation and environmental influences jointly contribute to the survival and development of organisms, encompassing both plants and animals. The environment plays a crucial role in shaping gene expression and phenotypic traits in these organisms [11], while gene regulation is central to the biological adaptation of organisms to environmental changes [12]. For example, certain genes, such as *OsGI*, *Hd1*, and *OsphyB*, participate in the regulation of the high adaptive potential exhibited by rice landraces in response to climate change [13]. The phenotypic variation in photosynthetic traits was also highly influenced by complex and diverse environmental conditions. Unfavorable environmental conditions, stress regulatory networks, and plant biochemical processes collectively constrain the photosynthetic efficiency of plants [14, 15]. By considering integrated environmental variables as an influential factor, the incorporation of these variables into the dynamic data of photosynthetic phenotypes presents a novel approach to unraveling the gene‒environmental regulatory mechanisms underlying genetic variation in photosynthetic phenotypes.

**Fig. 1:**
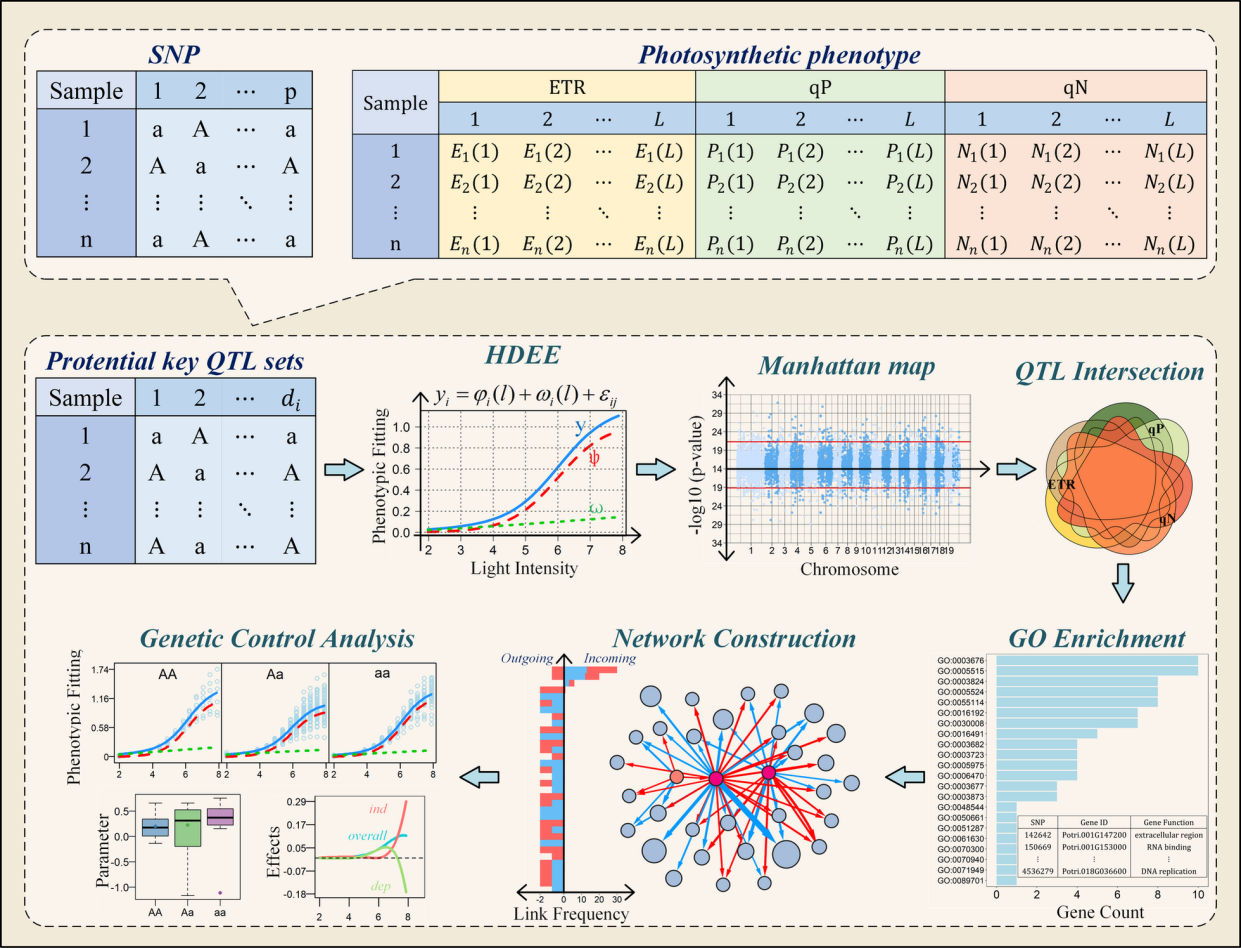
Schematic diagram of the model framework. It is applied to high-dimensional photosynthetic phenotypic data by constructing a set of potential key QTL sets to identify the key loci that regulate the changes in photosynthetic phenotypes under gradient light intensity to explore the mechanism of phenotypic response to different light gradients

In this study, we developed a comprehensive framework that integrates functional mapping [16–19], dynamic screening of gene‒environment complex systems, and differential networks based on evolutionary game theory [20] to investigate the dynamic genetic mechanisms underlying multiple phenotypes in response to the influence of integrated environmental variables. In this study, we investigated a natural population of *Populus simonii* as a case study. We employed optimal function mapping using the maximum likelihood method to identify genome-wide genetic loci associated with three key phenotypic traits, electron transfer rates (ETR), photochemical quenching (qP), and nonphotochemical quenching (qN), in photosystem II and established a set of candidate loci obtained through preliminary screening, referred to as “potential key QTL sets.” For the loci that potentially contribute to the dynamic changes in photosynthetic phenotypes in response to light intensity, we employed high-dimensional equations with environmental regulators (HDEE) based on a mapping approach to identify the dominant quantitative trait loci (QTLs) governing the photosystem. Additionally, we investigated the influence of environmental factors on different genotypes within the population by analyzing the mode of environmental effects. Furthermore, by constructing gene networks and enriching gene functions, we classified and delineated the roles of these loci. The schematic diagram of the model framework implemented in this paper is shown in Fig. 1. On the one hand, we revealed the epistatic relationship among significant gene loci involved in regulating photosynthetic phenotypes in response to light changes. On the other hand, key QTLs affecting multiple phenotypes were identified to investigate their pleiotropic expression in the photosynthetic system. This study employed statistical and computational methods to visually uncover the genetic mechanisms of photosynthesis, providing a feasible approach to elucidate the genetic plasticity of high-dimensional phenotype systems under dynamic environmental conditions.

## Results

### Construction and fitting of the HDEE

**Fig. 2:**
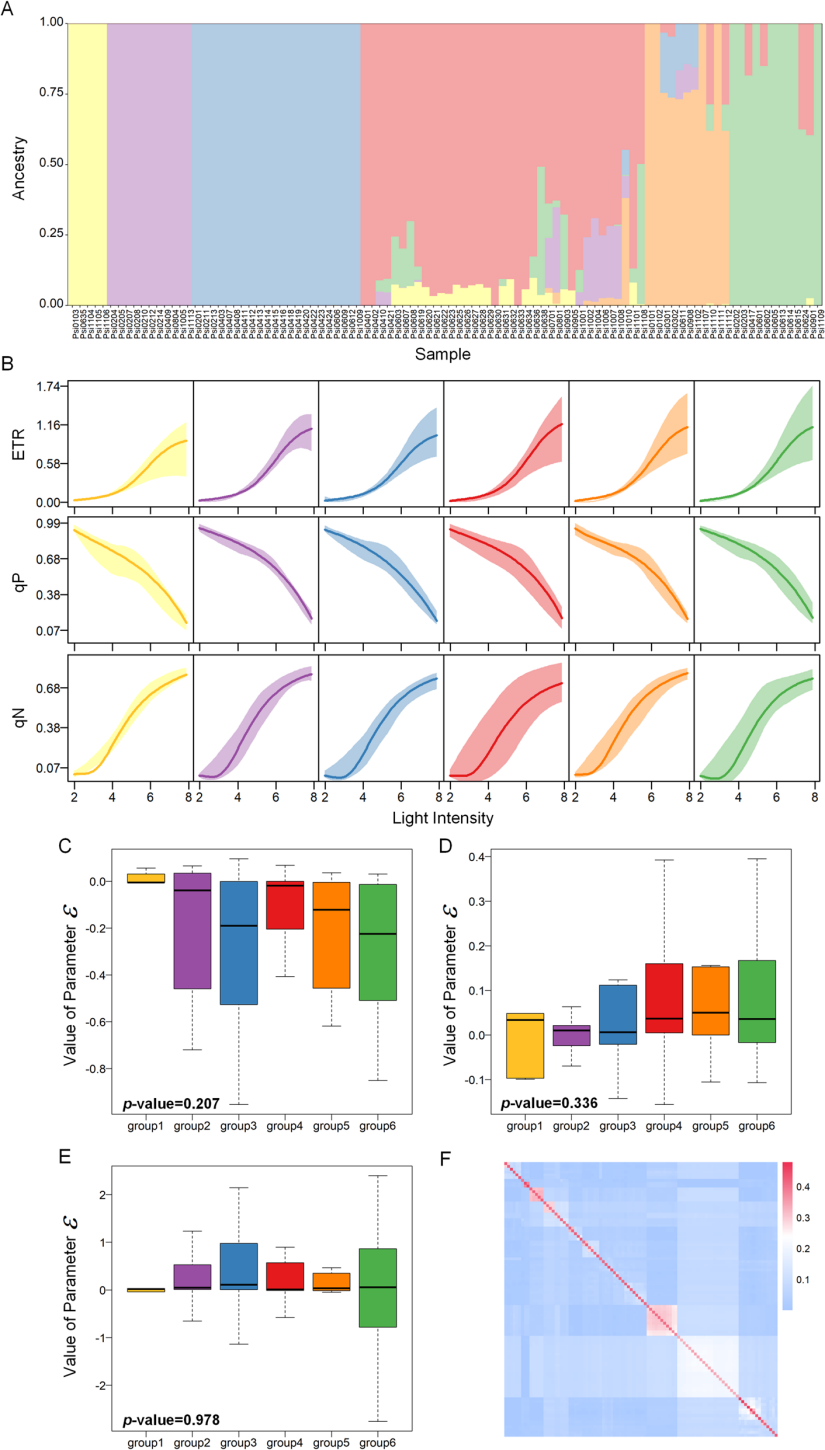
Population structure and kinship analysis of 98 samples of *Populs simowii*. **A** The population structure of 98 samples. Each color represents a group. Each column represents a sample, and each colored part in each column represents the proportion of the contribution of the population to the samples. **B** The photosynthetic phenotypic dynamics under gradient light intensities of ETR, qP, and qN of different groups. **C** Box plots illustrating the values of the population structure disturbance term of ETR, along with the *p*-value for the median from the Kruskal-Wallis test, are presented in the figure. **D** Box plots illustrating the values of the population structure disturbance term of qP, along with the *p*-value for the median from the Kruskal-Wallis test, are presented in the figure. **E** Box plots illustrating the values of the population structure disturbance term of qN, along with the *p*-value for the median from the Kruskal-Wallis test, are presented in the figure. **F** Heatmaps of the correlation coefficient of kinship for 98 samples

We decompose photosynthetic phenotype under gradient light into autogenic regulation terms ${\phi }_{i}\left(l\right)$ and environmental disturbance terms ${\omega }_{i}\left(l\right)$, as well as the perturbation parameters ${\epsilon }_{ij}$ for different populations of samples. The dynamic variation in the photosynthetic phenotype with light intensity, represented by ${\phi }_{i}\left(l\right)$, follows an S-shaped pattern, which can be effectively modeled using a growth curve, such as the Gompertz [21] and Logistic [22] curves, among others. The selection of the optimal expression of ${\phi }_{i}\left(l\right)$ is based on the numerical experimental results of phenotype fitting, including standard error, goodness of fit, and the comparative implementation of three information criteria (S Table 1, Additional file 2). In this study, the dataset of 98 samples was divided into 6 groups using Admixture software, as depicted in Fig. 2A, and the difference curves of photosynthetic phenotypes among the 6 groups with varying light intensities are presented in Fig. 2B. The trajectories of phenotypes with varying light intensities reveal a consistent average dynamic trend among different groups. The differences are primarily observed in the variance of the group phenotypes. Figure 2C-E show the distribution of group disturbance parameter values, providing a more intuitive observation that despite variations in the overall distribution range of group disturbance parameters, the median among different groups are highly similar. This similarity is further supported by Kruskal-Wallis tests with *p*-value greater than 0.05. The results of variance analysis for the parameters of the three phenotypes across different groups indicated nonsignificant population differences, as evidenced by *p*-values much higher than 0.05 in S Table 2 (see Additional file 2). Furthermore, we conducted an analysis of the genetic relationships among all samples (Fig. 2F) and determined that the correlation coefficient of the genetic relationships ranged from 0 to 0.48. Moreover, it was observed that more than 98.46% of the genetic relationships had correlation coefficients below 0.3. Based on these findings, it can be inferred that the impact of kinship within the natural population studied is relatively weak. In this study, the impact of population structure differences and genetic relationships among samples on phenotypic data was found to be negligible. Therefore, the disturbance term associated with population structure was omitted from the model. Combined with the results of the numerical analysis in S Table 1, high-dimensional equations with environmental regulators (HDEE) can be expressed as (See the Materials and Methods for details):

1 $$\left\{\begin{array}{l}y_1=\;\frac{K_1}{1+\;a_1e^{-b_1\mathit l}}\;+\mathit\;c_1l^{d_1}\\y_2=\;K_2\;\left(1-a_2e^{\mathit-{\mathit b}_2\mathit l}\right)\;+\;c_2l^{d_2}\\y_3=\;K_3e^{\mathit-\frac{\mathit a\mathit3}{\mathit l^{\mathit b\mathit3}}}\mathit\;+\;c_3l^{d_3}\end{array}\right.$$

**Fig. 3:**
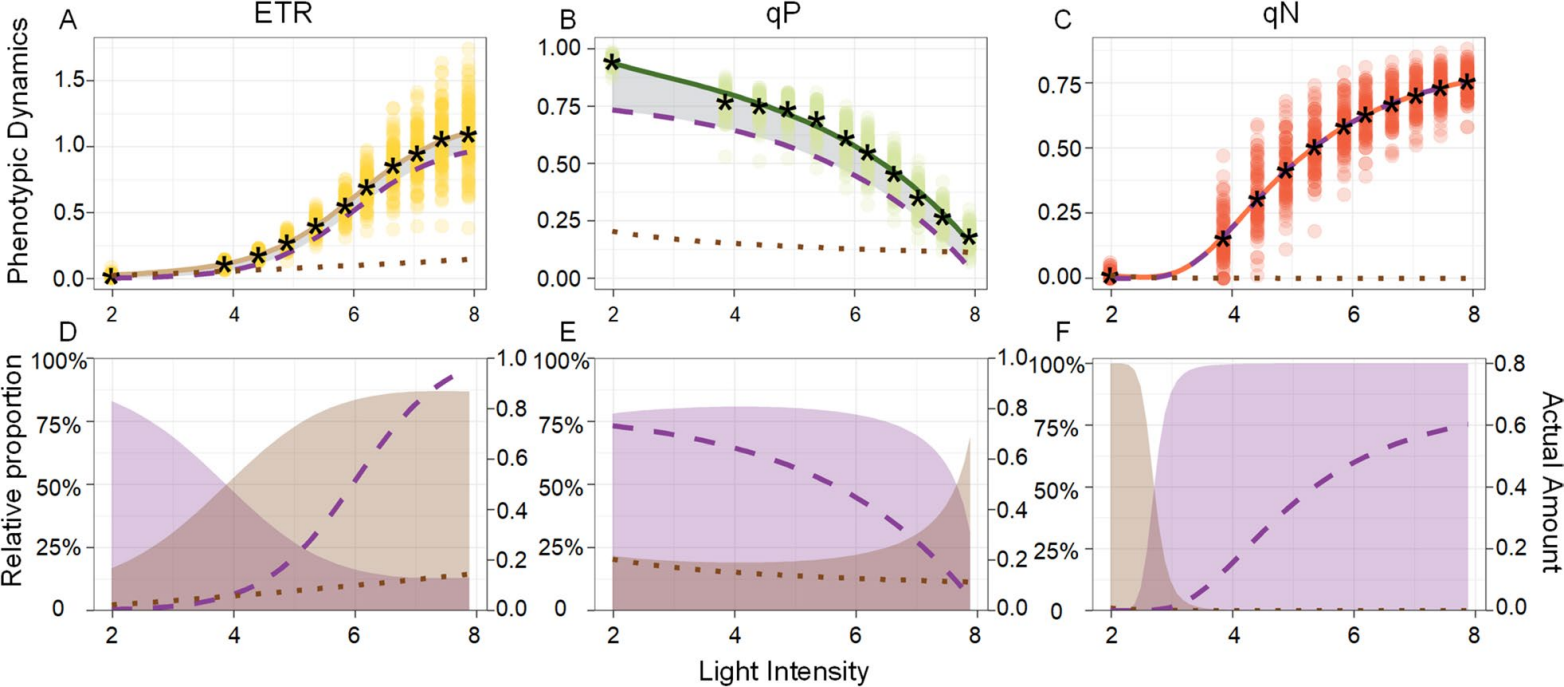
Photosynthetic phenotypic dynamics and component proportions. **A** HDEE fitting curve of phenotypic ETR, which is decomposed into an autogenic regulation curve (purple dashed line) and an environmental disturbance curve (brown dotted line). The observed phenotypic ranges of all samples are indicated by light yellow dots. **B** HDEE fitting curve of phenotypic qP. The observed phenotypic ranges of all samples are indicated by light green dots. **C** HDEE fitting curve of phenotypic qN. The observed phenotypic ranges of all samples are indicated by light orange dots. **D** The proportion of the autogenic regulation term (purple shaded portion) and environmental disturbance term (brown shaded portion) in overall ETR phenotypic dynamics. The lines represent the actual phenotypic values of autogenic dynamics and environmental interactions. **E** The proportion of the autogenic regulation term and environmental disturbance term in overall qP phenotypic dynamics. **F** The proportion of the autogenic regulation term and environmental disturbance term in overall qN phenotypic dynamics

S Table 1 illustrates that the equations incorporating the environmental disturbance term provide a more precise characterization of the changing patterns observed in the growth and development of the three photosynthetic phenotypes compared to the classical growth equations. Figure 3A-C depict scatter plots illustrating the variation in mean values for the three phenotypes in response to changes in gradient light intensity. The overall fitted curve, obtained using the HDEE model, closely aligns with the observed mean values of the actual phenotype, indicating a strong correspondence between the model and the real data. The independent growth curves and the environmental disturbance curves elucidate the dynamics of photosynthetic phenotypes and the variation attributed to environmental disturbance during the gradient light intensity change process. As light intensity increased, the phenotypic values of electron transfer rates (ETR) and nonphotochemical quenching (qN) exhibited a gradual increase, whereas the value of photochemical quenching (qP) decreased. The corresponding independent growth curves consistently followed this trend. However, the trend of the environmental disturbance curves did not entirely align with that of the overall phenotype. While the environmental disturbance showed a promoting effect on the increase in phenotypic values, the environmental disturbance of ETR increased with light intensity, the environmental disturbance of qP decreased with light intensity, and the environmental disturbance of qN tended to approach zero.

In addition, Fig. 3D-F show the proportion of independent growth curves and environmental disturbance curves in the overall phenotype. It can be observed that while the independent growth of ETR exhibits an upward trend with increasing light intensity, its relative proportion in the overall dynamics gradually decreases, while the proportion of environmental disturbance dynamics gradually increases. This indicates that the influence of light intensity gradually enhances the role of “environmental disturbance” in the photosynthetic phenotype. For qP, its environmental disturbance curve shows a downward trend, but its proportion in the overall phenotype is gradually increasing, indicating that the impact of environmental interference on qP is also gradually increasing. Although the disturbance curve of qN is approximately 0, the scale diagram reveals that at low light intensities (approximately 2), the proportion of environmental interaction components is 100%, but the proportion gradually decreases to 0 when the value reaches approximately 3. This observation indicates that qN is weakly influenced by the environment under conditions of high light intensity. The intensity of environmental disturbance is clearly influenced by light intensity, and the dynamic changes in phenotypic autogenic regulation in response to gradient light intensity may be influenced by genetic regulation.

### Identification of QTLs for high-dimensional photosynthetic system

**Fig. 4:**
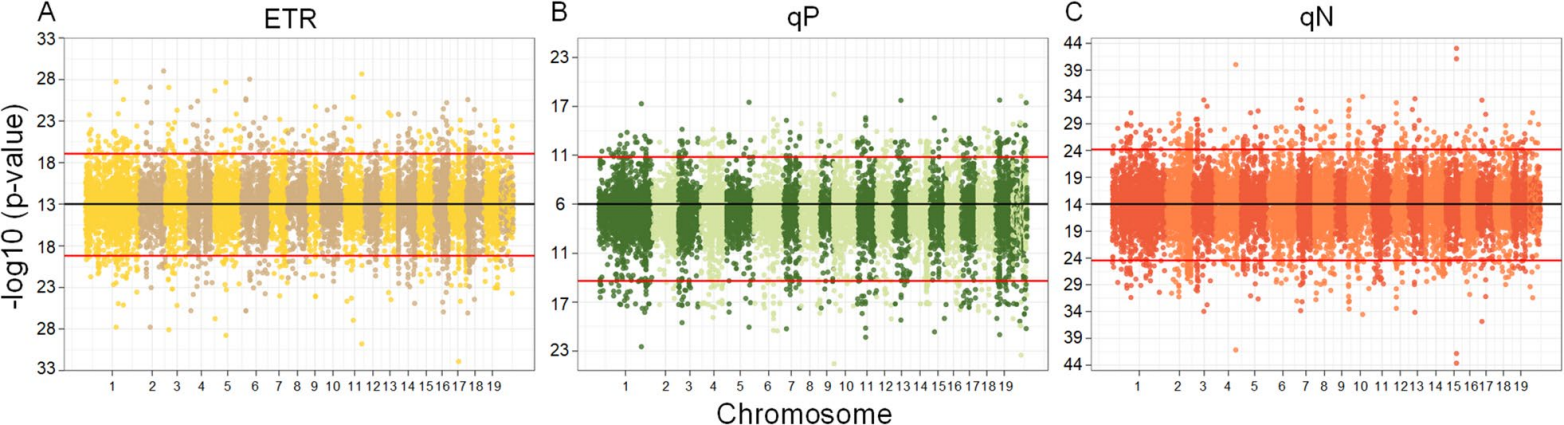
Manhattan plots of p values across 19 chromosomes of the *Populus* genome. **A** Test statistic values of SNPs were calculated by functional mapping based on the classical growth model (the upper panel) and HDEE (the lower panel) for the photosynthetic phenotypes of ETR. **B** Test statistic values of SNPs for the photosynthetic phenotypes of qP. **C** Test statistic values of SNPs for the photosynthetic phenotypes of qN. Red horizontal lines are the critical thresholds at the top 5% significance levels of potential key QTL sets

In the quantitative trait locus (QTL) mapping analysis, we initially conducted a genome-wide association analysis on 4,996,309 high-quality Single Nucleotide Polymorphism (SNP) data using the functional mapping approach with several classical equations (specifically focusing on the autogenic regulators in Eq. 1). This analysis led to the identification of 10,000 SNP loci as potential key QTL sets, which were considered to have the highest likelihood of influencing the photosynthetic phenotype under investigation. The histogram in S Fig. 1 (see Additional file 1) and the results of the Kolmogorov-Smirnov test indicated that the gradient light intensity and the average photosynthetic phenotype followed an approximately normal distribution. For the loci with high probabilities of influencing the dynamic process of photosynthetic phenotypes in response to light intensity, we employed static methods to identify sites that significantly regulate photosynthetic phenotypes across 11 light intensity groups, as depicted in S Fig. 2 (see Additional file 1). The identified gene loci have a significant impact on the variation in photosynthetic phenotypes under varying light intensities, and the gradual increase in light intensity results in alterations in the number and pathways of associated genetic loci involved in phenotypic plasticity. To investigate how significant QTLs regulate dynamic changes in phenotypes under gradient light intensity, we performed a high-dimensional phenotype-genotype association analysis based on HDEE. This analysis allowed us to identify the top 3% of significant quantitative trait loci associated with the three phenotypes, as illustrated in Fig. 4. Based on the autoregulatory growth model, which is represented by the classical growth equation, we found that in the association analysis of ETR, qP, and qN, 127 (42.33%), 96 (32.00%), and 76 (25.33%) of the significant sites, respectively, were annotated to genes with known functions in *Populus trichocarpa*. By utilizing HDEE for association analysis, we identified 129 (43.00%), 101 (33.67%), and 71 (23.67%) SNPs within genes of known function, respectively.

**Fig. 5:**
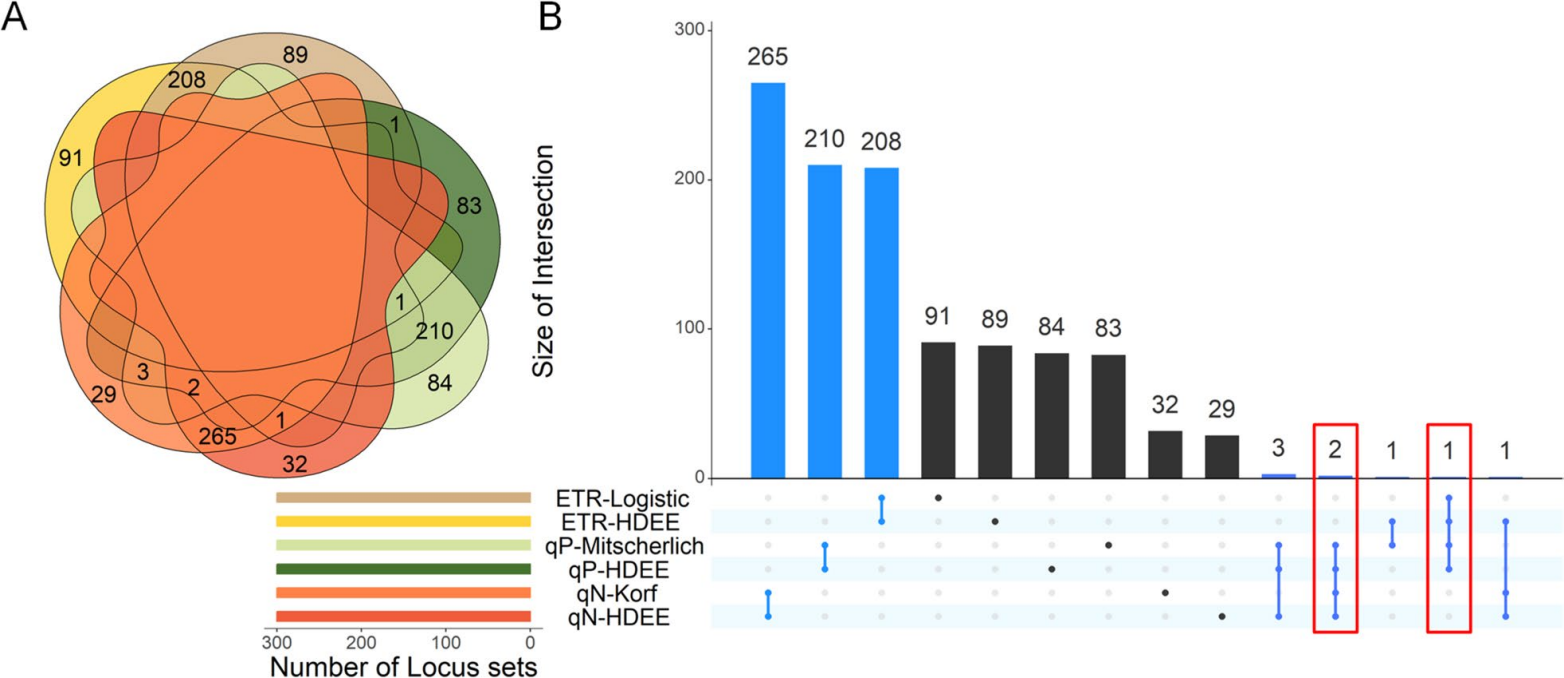
Comparative analysis of significant loci based on different growth models. **A** Venn plot of intersections of significant loci. **B** UpSet plot of the intersection of significant loci, in which the red boxes indicate the number of loci that have significant effects on two or more phenotypes

In the comparative analysis of significant loci identified using the autoregulatory growth model and HDEE (Fig. 5), we specifically emphasize loci that exhibit significant effects in both models. This is important because it allows us to mitigate the potential presence of false positives in the QTL mapping results. The loci that exhibit significance in both models simultaneously are considered to have higher reliability. These shared loci play a crucial genetic role in the genetic variation of the corresponding photosynthetic phenotype, exerting a strong and significant effect on the associated phenotype. Furthermore, these loci were identified through screening with HDEE, incorporating environmental regulators. This suggests that the regulation of gene loci in phenotypic expression may be influenced, to some extent, by environmental perturbations in addition to autologous growth regulation. For the ETR, qP, and qN phenotypes, we identified 209, 216, and 268 significant loci, respectively, that regulate photosynthetic phenotypes in response to changes in gradient light intensity. S Figs. 3, 4 and 5 displays the results of the gene ontology (GO) function enrichment analysis for the genes associated with these identified loci (see Additional file 1). Specifically, for the ETR phenotype in response to changes in light intensity, the top three enriched functions of the regulating genes include biological processes such as nucleic acid binding and protein binding, along with a molecular function related to catalytic activity. Significant sites that regulate the dynamics of qP phenotypes exhibit important functions such as Adenosine Triphosphate (ATP) binding and protein phosphorylation in biological processes, along with the molecular function of protein kinase activity. Similarly, significant gene functions at prominent sites associated with qN phenotypes also involve ATP binding, protein kinase activity, and protein phosphorylation.

Moreover, within the intersection of these pairs of significant loci, several noteworthy QTLs were found to regulate the growth and development of two phenotypes simultaneously (highlighted in the red box in Fig. 5B). Specifically, SNPs 2309065 and 3844928 influence the changes in both qP and qN in response to light intensity. Additionally, SNP 2443013 exerts an impact on both ETR and qP dynamics under varying light intensities. The pleiotropic effects of these loci are believed to be significant in the context of high-dimensional photosynthetic phenotypic systems.

### QTL network construction for photosynthetic phenotypes

**Fig. 6:**
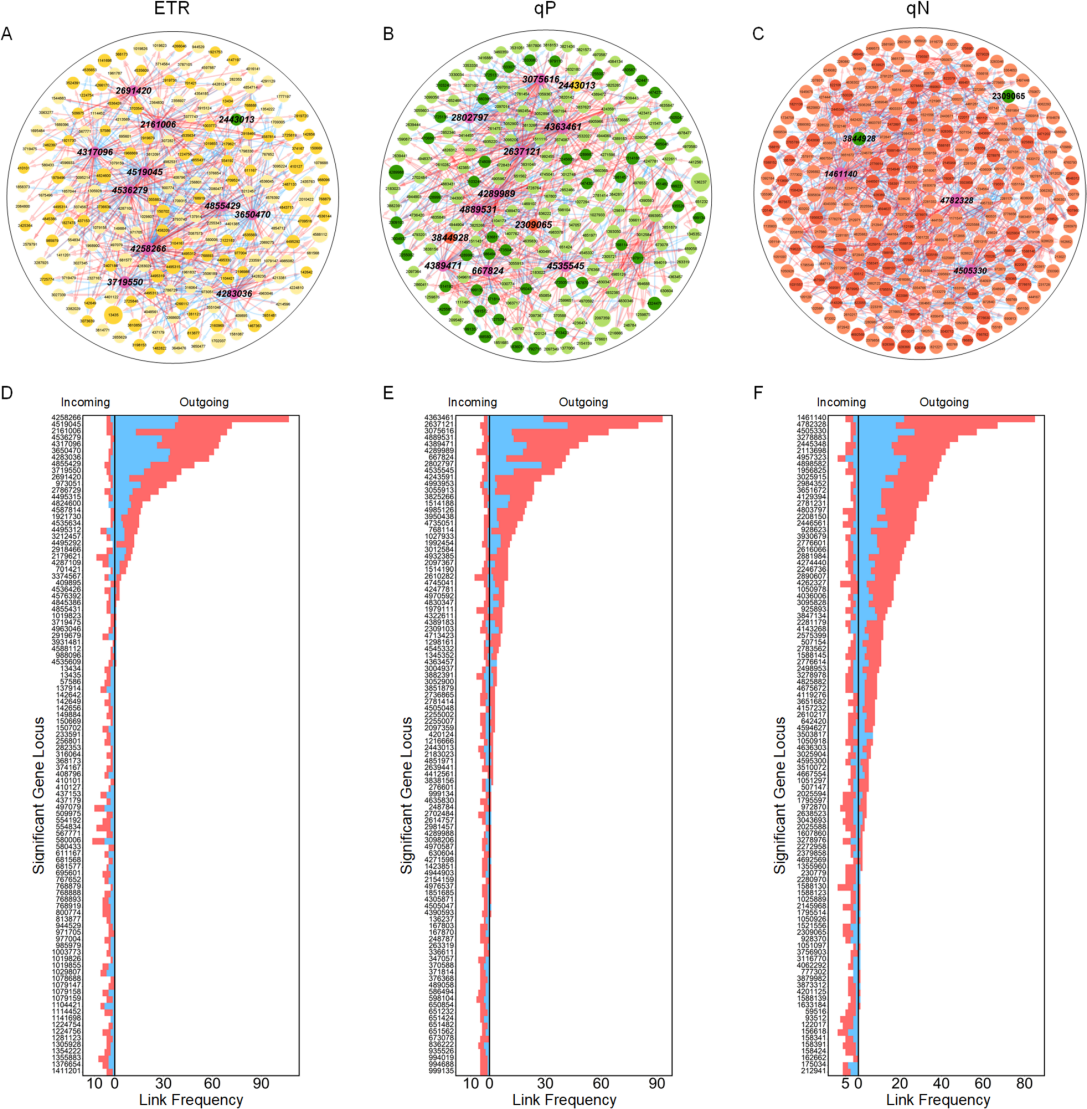
Genetic regulatory network analysis of significant SNPs. **A** Network structure of 209 significant SNPs with respect to the variation in ETR with gradient light intensity. **B** Network structure of 216 significant SNPs with respect to the variation in qP with gradient light intensity. **C** Network structure of 268 significant SNPs with respect to the variation in qN with gradient light intensity. The arrow indicates the direction in which one SNP activates (red arrow) or inhibits (blue arrow) another SNP. Highlighted pink nodes represent the hubs of the network. The size of the node represents the size of its own independent effect average. **D** Distribution of the number of outgoing and incoming links over 100 chosen nodes in the ETR network. **E** Distribution of the number of outgoing and incoming links over 100 chosen nodes in the qP network. **F** Distribution of the number of outgoing and incoming links over 100 chosen nodes in the qN network. The red and blue cumulative bars correspond to the arrows of the coincident colors in networks

The overlapping QTLs between the significant loci identified using the autogenous regulatory growth model and those identified using HDEE demonstrate significant regulatory effects on the associated photosynthetic phenotypes. Furthermore, there exist interactive relationships among the effects of these QTLs on phenotypes during adaptation to varying light intensities across gradients. Utilizing the dynamic curve of genetic effects of QTLs governing phenotypic changes, we established genetic regulatory networks among QTLs based on the principles of differential evolutionary game theory and modeled how the genetic regulation of a significant QTL on phenotype is influenced by its inherent control and the strategies employed by other QTLs (Fig. 6A-C). In the context of a network node A, an outgoing link refers to a link originating from node A and pointing toward other nodes. Conversely, an incoming link of node A denotes a link directed toward node A from another node. Based on the statistical data depicted in Fig. 6D-F, it is evident that despite the potentially large number of nodes within the network structure, each node is primarily influenced by only a limited number of other nodes in the network. Specifically, the number of incoming links between nodes tends to concentrate within the range of 1–13. This observation provides empirical evidence confirming the network sparsity principle within the genetic regulatory network that governs the photosynthetic phenotype. Nevertheless, there is substantial variation in the number of outgoing links among different nodes, ranging from 0 to 107, with only a few nodes exhibiting a notably high number of outgoing links. In the global network structure, nodes characterized by a significant number of outgoing links are referred to as network hubs. In this study, the designation of hubs is attributed to nodes with outgoing link counts exceeding 20% of the total number of nodes in the network. Given the robust regulatory influence exerted by hubs on other nodes within the network, they assume a crucial role in the dynamic manifestation of photosynthetic phenotypes.

 Within the ETR QTL network structure, a mere 865 links (1.99% of all possible links among 209 nodes) were identified. Among these links, 58.15% were determined to be positive, indicating their promotion of regulatory interactions, while the remaining 41.85% were classified as inhibitory links. The relationship observed among genetic loci governing ETR in response to varying light intensity primarily exhibited a cooperative nature. The network is distinguished by the presence of hubs, namely, SNP 4258266, SNP 4519045, SNP 2161006, SNP 4536279, SNP 4317096, SNP 3650470, SNP 4283036, SNP 4855429, SNP 3719550, and SNP 2691420. Based on gene function annotation results, it was determined that SNP 4519045 is positioned within the gene Potri.018G018800, which exhibits the function of 3*-hydroxyisobutyryl CoA hydrolase* activity (GO:0003860) in *Populus trichocarpa*. This specific site corresponds to the protein family *Enoyl-Coa hydratase/isomerase* (PF16113). Furthermore, SNP 4536279 is situated within the Potri.018 G036600, SNP 4317096 is located within the Potri.017 G017000, and SNP 4855429 is positioned within the Potri.019 G098500. Additionally, SNP 3719550 is located within the Potri.014 G118100, and SNP 2691420 is positioned within the Potri.009 G023800. For detailed information regarding the specific functions of genes in *Populus trichocarpa* and the homologous genes in Arabidopsis thaliana, please refer to S Table 3 (see Additional file 2).

**Fig. 7:**
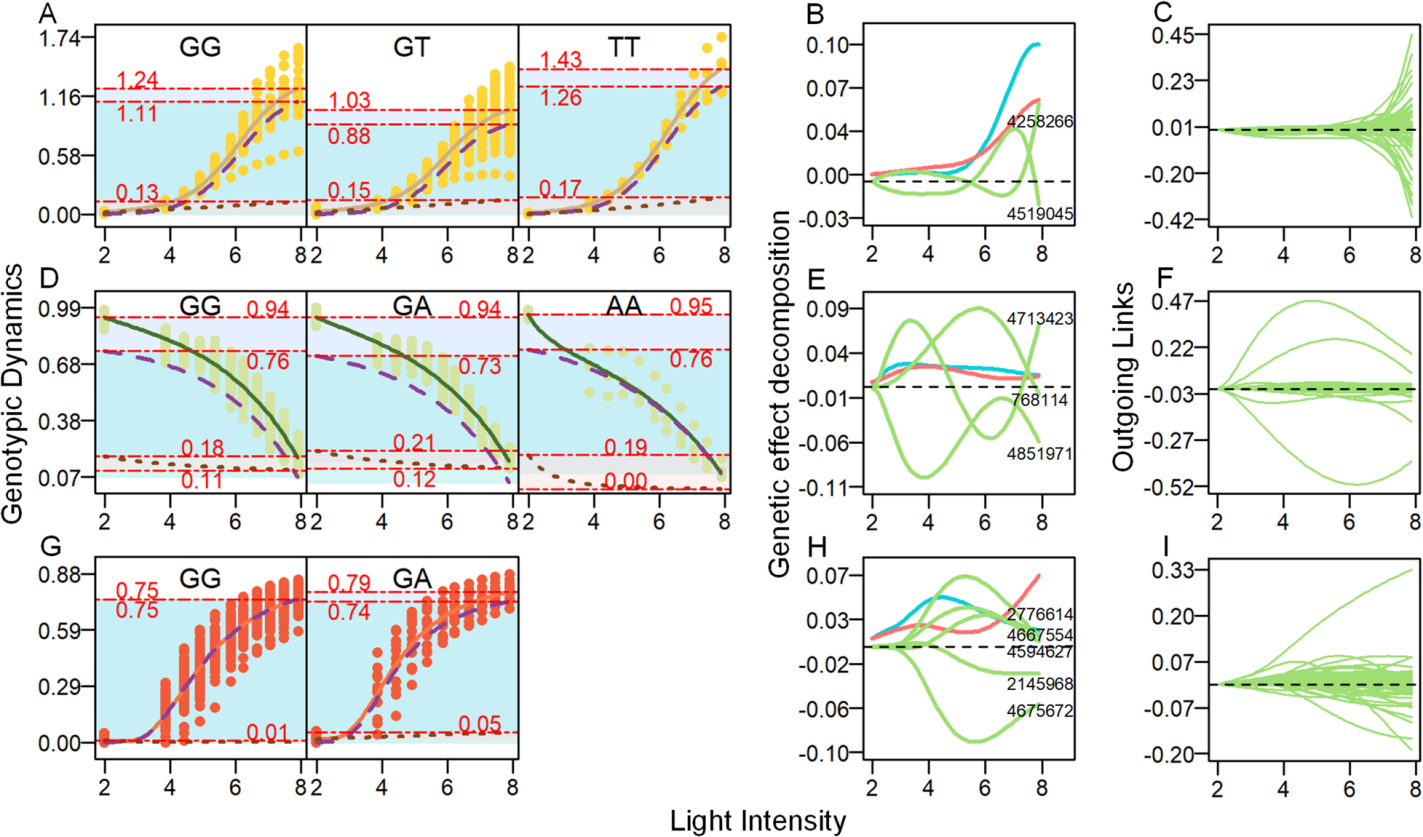
Analysis of genetic control of phenotypic dynamics by hub nodes. **A** ETR dynamic curves of different genotypes for SNP 4536279. **B** Curves of genetic effects on the phenotype of ETR for SNP 4536279. The overall genetic effect (blue line) is decomposed into independent effects (red line) and dependent effects (green line) due to regulation by other SNPs. **C** All dependent curves of SNP 4536279’s action on other SNPs in the ETR network. **D** qP dynamic curves of different genotypes of SNP 4535545. **E** Curves of genetic effects on the phenotype of qP for SNP 4535545. **F** All dependent curves of SNP 4535545’s action on other SNPs in the qP network. **G** qN dynamic curves of different genotypes of SNP 1461140. **H** Curves of genetic effects on the phenotype of qN for SNP 1461140. **I** All dependent curves of SNP 1461140’s action on other SNPs in the qN network

Individuals harboring different genotypes can exhibit varying responses to environmental fluctuations, resulting in diverse phenotypic landscapes. To investigate the specific genetic structure underlying the regulatory changes in phenotypic traits, we focused on an intercross locus, namely, SNP 4536279, which was selected from the hub node within the ETR network. Based on the analysis of genotypic difference curves (Fig. 7A) for SNP 4536279, it was observed that samples with the TT genotype exhibited higher phenotypic values (asymptotic growth value) of ETR with increasing light intensity, followed by samples with the GG genotype and then the GT genotype. The independent curves showed consistent differences in line with these observations. The sample corresponding to the TT genotype exhibited a greater environmental disturbance term, while the perturbation term associated with the GT genotype was larger than that associated with the GG genotype. These findings suggest that although the sample with the GT genotype had a lower ETR value, it demonstrated a higher proportion of regulation in response to the light environment.

The disparity in phenotypic values among different genotypes represents the manifestation of the genetic effect of the QTL on the phenotype. From Fig. 7B, it is evident that the overall genetic effect value of SNP 4536279 exhibits an upward trend as the light intensity increases. This indicates that the genetic regulation of genes on the ETR response to changes in light intensity intensifies with higher light intensity. The curves depicting independent genetic effects displayed similar trends as the light intensity changed. However, when the light intensity reached 6, the value of independent genetic effects was notably lower than that of the overall genetic effects. The higher overall genetic effect of SNP 4536279 can be attributed to the promotion exerted by SNP 4258266 and SNP 4519045, specifically within the Potri.018 G018800. Interestingly, based on the observed trend in the effect curve, it can be noted that the promoting effect of SNP 4519045 gradually diminished after reaching a light intensity of 7, eventually even transitioning into a negative value, indicating an inhibitory role. Conversely, the promoting effect of SNP 4258266 demonstrated a gradual increase after the light intensity reached 7. Consequently, it is plausible to suggest that the effects of these two sites on SNP 4536279 may exhibit mutual exclusivity.

As a hub node that assumes a dominant regulatory role within the network, our primary focus lies in understanding how this particular site regulates other sites. Based on the findings depicted in Fig. 7C, SNP 4536279 exerts a regulatory effect on 65 nodes. Among these nodes, the promotion effect accounts for 55.38%, while the inhibition effect accounts for 44.62%. The intensity of regulation remained close to 0 within the light intensity range of 2–6. However, as the light intensity continued to increase, the regulatory effect significantly intensified. Notably, the most pronounced regulation occurred when the promotion effect on SNP 142656 reached 0.45 and the inhibition effect on SNP 150702 reached 0.41, both observed at a light intensity of 8.

Within the genetic regulatory network of qP, a total of 908 links were identified, which corresponds to 1.96% of all potential pairwise regulations. Among these links, approximately 66.96% were found to have a positive regulatory role, while approximately 33.04% exhibited a negative regulatory role. Cooperation emerged as the primary interaction strategy among the genetic loci involved in regulating qP changes in response to varying light intensity gradients. The hub nodes in the qP network, characterized by having the number of outgoing links surpassing 20% of the total number of nodes, encompass SNP 4363461, SNP 2637121, SNP 3075616, SNP 4889531, SNP 4389471, SNP 4289989, SNP 667824, SNP 2802797, and SNP 4535545. SNP 4289989 is situated within the candidate gene Potri.016 G130600, which exhibits GTPase activity (GO:0003924) and GTP binding (GO:0005525). Additionally, according to the Protein FAMilies database (Pfam) annotation, it belongs to the Ras family (PF00071), a significant regulator involved in vesicle formation, motility, and fusion. We conducted an analysis of the genotypic curves (Fig. 7D) associated with the hub locus responsible for regulating the photosynthetic phenotype qP in response to varying light intensity gradients. Our findings revealed that environmental interference under different genotypes consistently exhibited a promotion effect on the qP phenotype as the light intensity gradient changed. However, it was observed that the promoting effect diminished with increasing light intensity. Additionally, it was found that the samples with genotype GA exhibited a higher degree of environmental disturbance.

Based on the analysis of the genetic effect of the hub SNP 4289989 (Fig. 7E), the effect curve exhibited an initial increase within the range of low light intensity (2–3) and subsequently decreased with the changing gradient light intensity. Moreover, the trend and variation range of the independent genetic effect curves were essentially similar to those of the overall curves. SNP 4535545 is promoted by SNP 768114 (Potri.002G095700) and inhibited by SNP 4851971. Additionally, the effect of SNP 4713423 (Potri.019G016100) exhibits a promotion trend within the range of 2–5, followed by inhibition within the range of 5–7 and subsequent promotion within the range of 7–8. In the overall network structure, SNP 4535545 exhibited regulatory effects on 35 SNPs, with the promoting effect accounting for 62.86% and the inhibiting effect accounting for 37.14% (Fig. 7F). The more significant regulatory effects of SNP 4535545 included the inhibition of SNP 2097359, which exhibited an increasing trend (reaching 0.51) within the light intensity range of 2–6, followed by a decrease. Additionally, there was promotion observed on SNP 4993953 and SNP 4851971, which initially increased and then decreased, with maximum values of 0.47 and 0.27, respectively.

In Fig. 4C, the genetic regulatory network of qN consists of 1250 links, which represents 1.75% of all possible relationships. Among these links, 770 indicate facilitation, accounting for 61.6% of the total, while 480 links exhibit inhibition, accounting for 38.4% of all existing relationships. Among them, SNP 1461140, SNP 4782328, and SNP 4505330 serve as hub nodes in the network. In this study, we conducted an analysis to investigate the genetic control of SNP 1461140 on the phenotypic trait qN. From the analysis of Fig. 7G, it is evident that environmental perturbations exhibit a mild promoting effect on the variation in the qN phenotype value under gradient light intensity. Notably, the samples with genotype GA demonstrate a stronger influence of environmental perturbations compared to the samples with genotype GG. The overall genetic effect curve exhibited an initial increase followed by a decrease, whereas the independent genetic effect curve generally showed an upward trend, with a slight decline observed between light intensities 4–6 (Fig. 7H). In the light intensity range from 2 to 6, the overall genetic effect surpassed the independent genetic effect, primarily due to the promotion effect of SNPs 2776614, 4667554, and 4594627 on the genetic control of phenotypic response to light intensity at this locus. Although SNP 1461140 plays a pivotal role in the genetic regulatory network of qP, its genetic control of the phenotype primarily arises from the promotion effect exerted by other SNPs. However, when the light intensity reached 7, the overall genetic effects became inferior to the independent genetic effects, mainly due to the suppression exerted by SNP 2145968 and SNP 4675672. We observed that although SNP 1461140 is not located within known genes, it is influenced by several loci within genes, including SNP 2776614 (Potri.010G001600), SNP 4594627 (Potri.018G060900), SNP 2145968 (Potri.006G199000), and SNP 4675672 (Potri.018G112200). The influence of these SNPs on SNP 1461140 may explain its significance as a central node in the network structure of qN. Additionally, we observed that the genetic effects of 63 other SNPs were transitively promoted, while the genetic effects of 22 SNPs were inhibited in the network (Fig. 7I). Notably, the promotion effect on SNP 212941 increased to 0.33 with the progressive rise in light intensity, which was considerably higher compared to other SNPs.

### Analysis of the pleiotropic effects of QTLs on multiple phenotypes

**Fig. 8:**
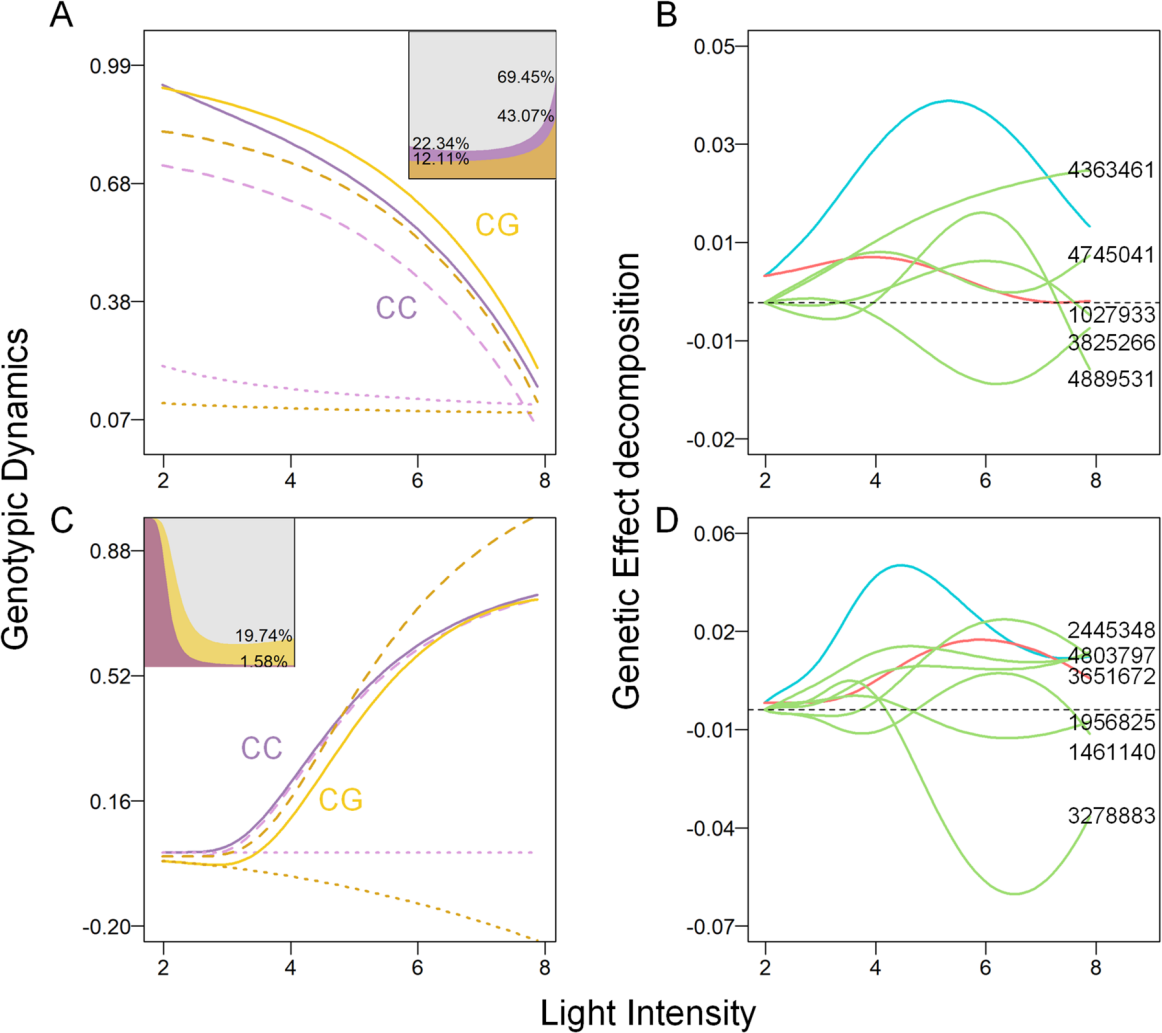
Genetic analysis of the significant SNP 2309065 regulating qP and qN. **A** Genotypic dynamic curves of qP. The picture in the upper right shows the proportion of the environmental disturbance term to the phenotype. **B** Genetic effect curve and decomposition in the qP network. **C** Genotypic dynamic curves of qN. The picture in the upper left shows the proportion of the environmental disturbance term to the phenotype. **D** Genetic effect curve and decomposition in the qN network

Based on the comparative analysis results presented in Fig. 5, it can be observed that certain loci within the high-dimensional photosynthetic phenotype system exhibit simultaneous regulation of multiple photosynthetic phenotypes in response to changes in gradient light intensity. This phenomenon indicates the presence of pleiotropic control, where gene regulation influences the expression of multiple phenotypic traits. We selected a specific locus, SNP 2309065, situated on chromosome 7, which exhibits an impact on the response of both qP and qN values to changes in light intensity. The objective of our analysis was to investigate the genetic control exerted by this locus on the two photosynthetic phenotypes. As depicted in Fig. 8A and C, it is evident that the overall qP curves, under the influence of SNP 2309065 genotypes, exhibit a higher magnitude compared to the independent dynamic curves. This observation suggests that the perturbation of the light environment positively influences the phenotype qP, with a more pronounced effect observed in samples with the CG genotype. Regarding the photosynthetic phenotype qN, the CG sample exhibited lower phenotypic values than the CC sample, but their values tended to converge under high light conditions. Specifically, the overall dynamic curve of the CC samples closely aligned with the independent curve, while the independent growth curve of the CG samples significantly surpassed the overall dynamic curve when exposed to high-intensity light perturbation. Notably, the magnitude of the perturbation term was positively correlated with the light intensity. Furthermore, as anticipated, the impact of light intensity perturbation on the overall photochemical quenching exhibited a progressive increase with rising light levels. In contrast, the proportion of the effect on nonphotochemical quenching gradually diminished. Among them, the proportion of light environment disturbance in qP was found to be higher in the CC genotype than in the CG genotype, accounting for 22.34% versus 12.11% under low light conditions and 69.45% versus 43.07% under high light conditions. In contrast, the proportion of the disturbance term in phenotypic qN was lower, at 1.58% versus 19.74%. These results indicate that *Populus simonii* individuals with the CC genotype at SNP 2309065 exhibit greater potential for photosynthetic activity. On the other hand, individuals with the CG genotype demonstrate a stronger ability to implement self-photoprotection through the dissipation of excess excitability via heat dissipation.

In the network structure (Fig. 8B and D), the independent genetic effects of SNP 2309065 on the regulation of qP and qN responses to light were found to be smaller than the overall genetic effects. However, as the light intensity increased, the independent effects on regulating qP gradually decreased, and the disparity between the independent effects and the overall effects increased. Under high light intensity, SNPs 4363461, 4745041, SNP 1027933, and 3825266 exhibit a shared positive promotion effect on the genetic regulation of the qP phenotype. However, the genetic control of the locus on the phenotype is reversely inhibited by SNP 3825266. In the qN network, the independent effect gradually increased, causing the overall effect curve to converge with the increase in light intensity despite the presence of regulatory effects at other sites. The promoting effects of SNP 2445348 (Potri.008G032901), SNP 4803797, SNP 3651672, and SNP 1461140, as well as the inhibiting effects of SNP 1956825 (Potri.006G097600) and SNP 3278883 (Potri.012G044701), offset each other.

**Fig. 9:**
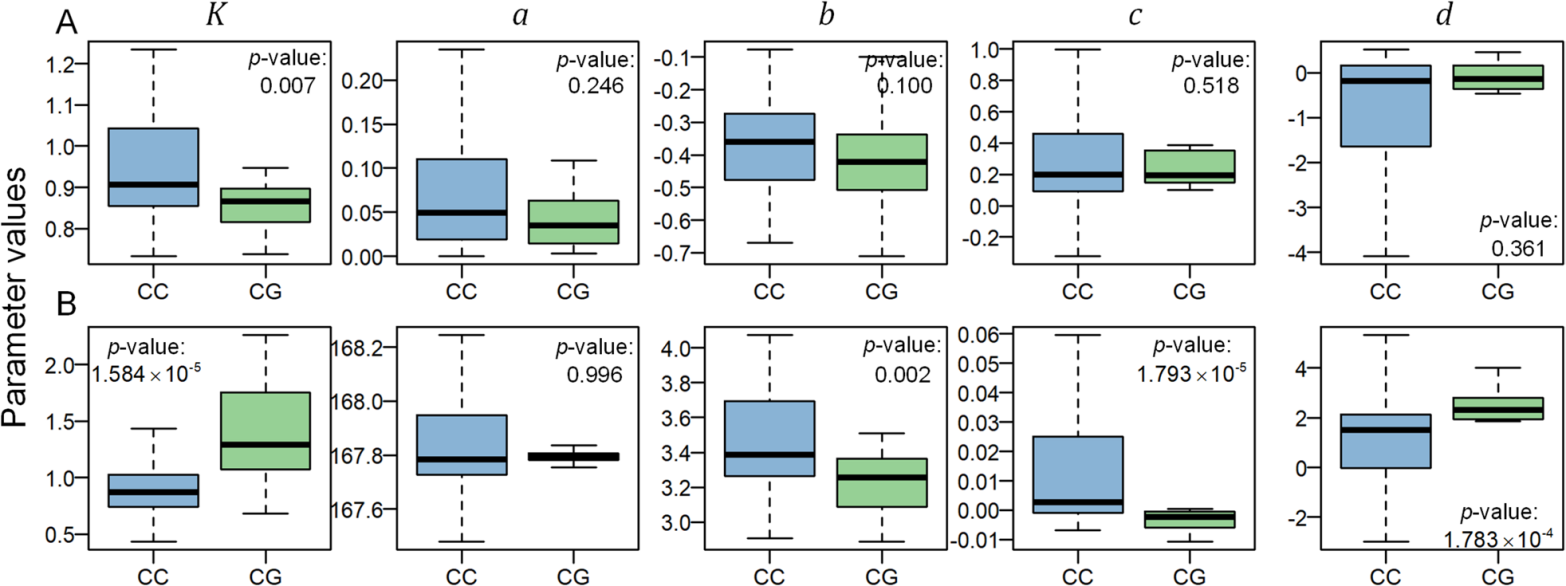
Box plots of the parameters of SNP 2309065 regulating qP and qN. **A** Genotype differences in HDEE parameters of qP. **B** Genotype differences in HDEE parameters of qN. The *p*-values for the medians from the Mann-Whitney U test are provided in the figures

Based on the quantile analysis of growth parameters of the phenotypic curves under different genotypes (Fig. 9), SNP 2,309,065 exhibited genetic control over the five growth parameters of qP and qN. However, distinctions were primarily observed in the regulation of autogenic regulation parameters’ distribution. In the regulation of phenotypic qP, the median of the asymptotic value (*K*) in the curves with increasing light intensity was smaller for the CG genotype than for the CC genotype (*p*-value = 0.007). For qN, genetic control is predominantly observed in the asymptotic value *K* (*p*-value = 1.584 × 10^−5^) and the parameter *b* (*p*-value = 0.002), which determines the position of the inflection point of the function. Notably, the genotype CG exhibits a larger *K* value, while the *b* value is smaller. The mean values of parameter *a*, which is associated with the relative growth rate of trees across different genotypes, exhibited minimal variation. However, the samples from genotype CG showed a highly concentrated distribution, whereas genotype CC samples were concentrated toward the higher end of the mean value.

The average values of the disturbance rate and disturbance scale coefficient in the environmental disturbance term, which are of particular interest, are comparable between the two genotypes. However, it is worth noting that the parameter distribution of the CC genotype is more dispersed for qP. This finding suggests that *Populus simonii* with the CC genotype at SNP 2309065 may exhibit a higher degree of responsiveness to environmental fluctuations. In terms of the parameters of phenotype qN, there is a more pronounced difference in the parameter distribution between the two genotypes. Specifically, the parameter *c* shows greater variability and tends to have larger values in the CC genotype samples (*p*-value = 1.793 × 10^−5^). Conversely, the parameter *d* exhibits higher variability and tends to have smaller values in the CC genotype samples (*p*-value = 1.783 × 10^−4^). The results of the parameter analysis indicated that *Populus simonii* with the CG genotype at SNP 2309065 exhibited greater stability in response to changes in the light environment.

## Discussion

Photosystem II (PSII) is a pigment-protein complex that facilitates the conversion of light energy into chemical energy, resulting in the liberation of dioxygen [23]. It functions as the exclusive enzyme responsible for light-induced water oxidation, a crucial process essential for the maintenance of life on our planet through photosynthesis [24–27]. Alterations in numerous photosynthetic processes, encompassing both light and dark reactions, exert feedback effects on photosystem II (PSII). These effects can be discerned through chlorophyll fluorescence, primarily originating from chlorophyll a within the PSII complex [28–31]. The phenotypes studied in this paper, namely, ETR (electron transport rate), qP (photochemical quenching), and qN (nonphotochemical quenching), are essential parameters in characterizing chlorophyll fluorescence kinetics. These parameters provide insights into the electron transfer process within PSII, photosynthetic activity, and photoprotection ability, respectively. In the process of plant growth and development, light intensity serves as a crucial environmental factor. The measured parameter values obtained under gradient light intensity conditions represent quantitative traits of plants and collectively form a high-dimensional photosynthetic phenotypic system. The response and adaptation of the plant photosynthetic system to changes in the light environment are controlled by genes involved in the photosynthetic pathway [32].

In this study, we integrated classical growth equations with environmental regulators to comprehensively characterize phenotypic changes. By employing high-dimensional equations, we can effectively dissect the photosynthetic phenotype into an autoregulatory component and an environmental disturbance component, thereby enabling a detailed analysis of the environmental response in photosynthetic traits. The findings from our study unveiled that despite the distinct patterns observed in the changes in ETR and qP phenotypic values with increasing light intensity, the proportion of environmental disturbance terms in both phenotypes exhibited an upward trend, indicating a growing impact of the light environment, particularly under high-light conditions. Conversely, for the qN phenotype, the influence of the light environment’s disturbance diminished as light intensity increased, suggesting that the greater dissipation of energy by plants through nonphotochemical processes resulted in a reduced influence of light intensity on this particular phenotype.

Photosynthesis, as a crucial life process, has been extensively studied to explore the genes involved in various plant pathways related to photosynthesis, with the goal of determining the genetic mechanisms of these plants. For instance, using composite interval mapping, QTLs for two photosynthetic stages in *Brassica napus* have been identified to explain the variation in photosynthetic phenotypes [33]. Hervé et al. identified QTLs for several physiological traits in sunflowers, providing the first description of genomic regions associated with sunflower yield [34]. And several putative QTLs related to rice photosynthesis and associated physiological traits have been detected [35, 36]. Under greenhouse conditions or different stress conditions, drought-tolerant wheat QTL [37], saline-tolerant rice QTL [38], and salt-tolerant barley QTL [39] related to photosynthetic traits have been identified. In *Populus simonii*, Deoxyribonucleic Acid (DNA) methylation has been identified to play a crucial role in leaf development and the regulation of photosynthesis [40]. However, to our knowledge, a comprehensive understanding of the genetic basis underlying the expression of complex traits during development requires detailed descriptions not only of their potential individual genes but also of their interactions as a whole. Although existing correlation analyses have achieved the construction of co-expression networks [41], our analytical approach in this study establishes a directed, weighted, and fully informative network, uncovering previously uncharacterized molecular mechanisms.

At the genome-wide level, we described the genetic variation and environmental response of chlorophyll fluorescence kinetic parameters by integrating mapping theory and multidisciplinary elements. We established a conceptual model framework by incorporating environmental considerations into the theoretical framework of functional mapping. Through comparative analysis with traditional equations, we identified significant QTLs associated with three important phenotypes. Moreover, through the construction of genetic networks using a differential equation model based on genetic effect values, we provided valuable insights into the genetic structure of photosynthetic phenotypic systems regulated by the detected QTLs responsible for photosynthetic phenotypes. The hub nodes within the network play a crucial role in shaping the genetic architecture of overall phenotype regulation, and some of them can annotate the important functions of the genes in which they are located. Using the genetic regulatory network of ETR as a case study, out of the 10 identified hub nodes, six nodes are linked to known functions. SNP 2691420 belongs to the gene Potri.009G023800 and is involved in protein dimerization activity (GO:0046983). SNP 3719550 corresponds to the gene Potri.014G118100 and is associated with zinc ion binding (GO:0008270) and nucleic acid binding (GO:0003676). SNP 4317096 is related to a lysine methyltransferase gene (PF10294) on Potri.017G017000. SNP 4519045 is situated within the gene Potri.018G018800 and is linked to *3-hydroxyisobutyryl-CoA hydrolase* activity (GO:0003860). SNP 4536279 is located in the Potri.018G036600 gene and exhibits multiple functions, including DNA-directed DNA polymerase activity (GO:0003887), DNA replication (GO:0006260), DNA-dependent DNA replication (GO:0006261), nucleic acid binding (GO:0003676), DNA binding (GO:0003677), and ATP binding (GO:0005524). Last, SNP 4855429 belongs to the gene Potri.019G098500, which is associated with ADP binding (GO:0043531), signal transduction (GO:0007165), and protein binding (GO:0005515). This observation underscores the crucial role of network hubs in governing the dynamic alterations observed in photosynthetic phenotypes. The GO analysis of the gene functions associated with the identified QTLs serves as a valuable basis for interpreting and validating the model results while also providing a novel perspective for exploring the potential regulation of photosynthesis.

In addition to the hub nodes within the networks, the network structure plays a crucial role in providing a comprehensive mechanism for understanding the interactions among genes and their collective regulation of complex phenotypes. This mechanism can be effectively visualized by decomposing the genetic effect curves, which exhibit variations in response to changes in light intensity. The statistical analysis of link distribution within the network, coupled with the identification and analysis of epistatic relationships among nodes, elucidated the control mechanisms of significant QTLs and their interactions, highlighting their influence on the plasticity of photosynthetic phenotypes across varying light intensities. We observed that the independent genetic effects of the majority of nodes in the network were smaller or comparable to the overall genetic effect, as illustrated by the genetic effect curves of SNP 2309065 depicted in Fig. 8B and D. However, it is important to highlight that certain noncentral nodes displayed prominent independent genetic effects but exhibited lower overall regulatory effects, owing to the inhibition exerted by other SNPs. For instance, in the effect decomposition curves of the 6 SNPs shown in S Fig. 6, these nodes are annotated within gene regions such as Potri.001G012900 and Potri.004G182100, which could potentially play a crucial regulatory role in the response of photosynthetic phenotypes to changes in the light environment if not constrained by inhibitory effects from other sites (see Additional file 1). Further exploration of the intricate interplay among significant sites in the networks and the experimental suppression of these inhibitory SNPs could potentially enhance the response mechanism of plant photosynthesis to light environments.

Gene pleiotropy, characterized by the influence of a single gene on multiple phenotypic traits, enables us to investigate the interrelationships among biological processes and the common genetic elements that underlie the formation and functioning of diverse traits [42]. An additional significant advantage of the analysis of genetic variation in photosynthetic phenotypes presented in this study is its ability to identify QTLs that govern multiple phenotypes. For instance, as illustrated in Fig. 8, SNP 2309065 exhibits regulatory effects on the dynamic changes in both qP and qP, two photosynthetic phenotypes. Notably, *Populus simonii* individuals carrying the CC genotype at this locus demonstrate a higher photosynthetic potential under high-intensity light conditions rather than a reduced energy loss through heat dissipation. These findings have important implications for improving breeding strategies and developing targeted interventions to enhance various photosynthetic traits. Furthermore, by identifying genes exhibiting pleiotropy, we can uncover the relationships and connections among different photosynthetic phenotypes, thereby shedding light on the underlying biological pathways and networks governing these diverse phenotypic traits. This knowledge contributes to a deeper understanding of the integrated regulatory mechanisms orchestrating various aspects of photosynthesis.

We employed high-dimensional equations incorporating environmental factors to investigate photosynthetic phenotypes, identified significant QTLs governing the regulation of photosynthetic parameters in response to varying light intensities, and developed a comprehensive model framework incorporating genetic regulatory networks. This integrated approach allowed us to gain insights into the genetic architecture underlying phenotypic variation and plasticity in photosynthesis. In the enhancement of agroforestry, the genetic variation of photosynthetic phenotypes can yield vital information for crop breeding [43]. The selection of varieties exhibiting strong adaptability to light environments holds the potential to enhance crop yield and stress resistance [37–39]. Additionally, this approach may contribute to the improvement of energy efficiency [44] and the exploration of novel bioenergy sources [1]. Simultaneously, comprehending the genetic adaptation of plants in diverse light environments aids in understanding their adaptive capacity to climate change. This understanding is pivotal for predicting the responses of plant communities and ecosystems to future climate changes, as well as for the management and conservation of ecosystems.

However, our model still has several limitations that can be addressed and improved upon in future research. First, as a conceptual model approach, we constructed potential sets of key QTLs using functional mapping based on classical equations to ensure computational efficiency. However, it is important to note that there may exist crucial loci in the whole genome that could be identified using HDEE-based mapping methods but may not be included in the set of results obtained from classical model mapping. Further investigations are required to verify this possibility in subsequent explorations. Second, it should be noted that the high-dimensional equation discussed in this study is in fact three-dimensional. The photosynthetic system encompasses other important parameters, such as the actual photoelectron yield of PSII and the instantaneous chlorophyll fluorescence intensity. To fully capture the comprehensive genetic mechanism underlying the function, dynamics, and evolution of the photosynthetic system under varying light intensities, a range of photosynthetic phenotypes need to be sampled. However, without the application of advanced spatial statistical methods, it becomes impractical to jointly model multiple phenotypes. Therefore, future research could benefit from the incorporation of sophisticated spatial statistical approaches to enhance the understanding of the complex interactions among various photosynthetic traits. In addition, several crucial environmental factors, including carbon dioxide concentration, temperature, and humidity, exert regulatory effects on the photosynthetic phenotype [8–10, 45]. However, since the phenotypes of the materials in this study were measured in a laboratory environment, factors influencing photosynthetic characteristics, apart from changes in light intensity, were not addressed in present study. Third, the identification of QTLs that govern multiple photosynthetic phenotypes suggests the existence of pleiotropic gene expression. As a single QTL can simultaneously influence multiple photosynthetic traits, there may be intricate interactions among various parameters within the photosynthetic system. For instance, Farooq et al. demonstrated that photosynthesis-related phenotypes, such as chlorophyll fluorescence kinetic parameters, exhibit a gradual dynamic equilibrium within photosystem II, wherein they coordinate and interact dynamically to optimize efficiency and mitigate photodamage [46]. This highlights the interconnectedness and coordinated regulation of multiple traits within the photosynthetic machinery. While these areas warrant further investigation, our model facilitates the analysis of the genetic architecture underlying the relationship between photosynthetic phenotypes and genotypes, holding biological significance and offering promising prospects for enhancing photosynthetic efficiency and guiding forestry production.

## Conclusions

In this article, we established high-dimensional equations incorporating environmental regulators to decompose the overall photosynthetic phenotype into autoregulatory and environmental disturbance components. We analyzed the proportion of light-induced disturbance in shaping the photosynthetic phenotype. The influence of light disturbance on the expression of ETR and qP phenotypes increased gradually with light intensity, showing a positive correlation with plant photosynthetic activity. Conversely, the proportion of environmental disturbance in the qN phenotype decreased with increasing light intensity, indicating an increased share of energy dissipated through heat as light intensity increased and a weakened effect of light on phenotypic regulation. The construction of the HDEE effectively elucidates the contribution of the light environment in shaping the photosynthetic phenotype under gradient light intensity.

We identified potential key QTL sets throughout the entire genome and subsequently localized QTLs that regulate the photosynthetic phenotype system, providing functional annotations. By analyzing the dynamic changes in genetic effects, we constructed an internal interaction network among QTLs, where competition or cooperation modes influenced the external regulation of genes on the photosynthetic phenotype. Notably, hub nodes exhibited greater importance in the network structure. Furthermore, our model framework successfully detected the expression of multiple photosynthetic phenotypes regulated by QTLs. The significant genotype differences observed in photosynthetic energy conversion and metabolic physiological processes hold guiding significance for the genetic breeding of trees aimed at improving photosynthetic efficiency.

## Materials and methods

### Plant materials

The experimental materials were collected between December 2015 and January 2016 from the natural distribution range of *Populus simonii*, spanning six provinces, autonomous regions, or municipalities directly under the Central Government in northern China, which includes Northwest China and North China. When selecting seeds from different individuals within each provenance, the spacing between individuals were maintained at a minimum of 50 cm. The sampled branches were stored in low-temperature stratified sand in a clonal arboretum of Guanxian County, Liaocheng City, Shandong Province (36°29′89″N, 115°27′34″E) for cutting propagation in the second year. One-year-old cutting seedlings with consistent growth were selected the following year and transplanted into 14 cm diameter and 12 cm height pots filled with a mixture of garden soil and matrix soil at a ratio of 1:3. The transplanted seedlings were then cultured under greenhouse conditions [47].

One week after planting, photosynthesis-related phenotypes of 120 *Populus simonii* were measured using chlorophyll fluorometers (PAM-2100). After a sufficient 15-minute dark adaptation of the leaves, 13 segments of excitation light with gradually increasing intensity were selected to illuminate the leaves, and the phenotype of chlorophyll fluorescence activated after each excitation light exposure was measured. A total of 12 phenotypic parameters, including light intensity, electron transport rate (ETR), photochemical quenching (qP), and s (qN), were measured. The temperature was consistently maintained at 24 °C with a standard deviation of 1 °C throughout the experiment. Humidity was kept within the range of 20–40% during measurements. In this study, ETR, qP and qN phenotypes of 98 samples were selected for analysis under 11 sets of light intensities, in which the variation range of light intensity was $7.243\mu\mathrm{mol}\cdot\;m^{\mathit-\mathit2}\mathit\cdot\mathit\;s^{\mathit-\mathit1}\;-\;2701.536\mu\mathrm{mol}\cdot\;m^{\mathit-\mathit2}\mathit\cdot\mathit\;s^{\mathit-\mathit1}\;$, and logarithmic treatment was adopted as the independent variable in this study.

The SNP data utilized in this study were obtained from the resequencing of immature leaves collected from *Populus simonii* populations, and the genomic DNA of the corresponding populations was extracted using the improved Cetyltrimethylammonium Bromide (CTAB) method. Population genome resequencing (> 15×) was conducted using the Illumina GA II sequencing platform. The raw data underwent quality control, and the filtered data were aligned to the *P. trichocarpa* reference genome (v3.0, https://phytozome-next.jgi.doe.gov/info/Ptrichocarpa_v3_0) using Burrows‒Wheeler alignment software (v0.7.5a-r405) with default parameters. The low-quality reads (sequencing quality score less than Q20) were filtered using Sequence Alignment/Map tools (SAMtools, v1.14) software. SNP identification was then performed using Genome Analysis Toolkit 4.0 (GATK4) with the following specific parameters: Variant Quality by Depth (QD) < 5.0 || Mapping Quality (MQ) < 40.0 || Fisher Strand Bias (FS) > 60.0 || Strand Odds Ratio (SOR) > 3.0 || Mapping Quality Rank Sum (MQRankSum) < -12.5 || Read Position Rank Sum (ReadPosRankSum) < -8.0. Finally, Variant Call Format tools (vcftools, v0.1.17) software was used to extract genome-wide SNPs, ensuring that the SNPs had two alleles and that the maximum missing rate per site was 20%. Additionally, the minimum minor allele frequency (MAF) was set to > 0.05. A total of 4,996,309 high-quality SNP sites were selected for subsequent analysis.

### Construction of the light intensity response function

The change in biological growth adheres to the fundamental principles of physics and development. However, the manifestation of specific traits is intricate and influenced by genetic factors, environmental regulatory factors, as well as endogenous and exogenous interactive factors [48]. In this study, we aimed to construct the genetic regulation mechanisms of three photosynthetic phenotypes, namely, ETR, qP, and qN, under comprehensive environmental disturbance using a systems theory approach. The variation in each phenotype can be decomposed into autogenic regulation terms ${\phi }_{i}\left(l\right)$ and environmental disturbance terms ${\omega }_{i}\left(l\right)$, and the perturbation parameters ${\epsilon }_{ij}$ of different populations of samples were introduced to represent the phenotype disturbance terms. The phenotypic response function to light intensity can be summarized as high-dimensional equations with environmental regulators (HDEE) in the following form:

2 $${y}_{i}={\phi }_{i}\left(l\right)+{\omega }_{i}\left(l\right)+{\epsilon }_{ij}$$

Here, the variable *l* represents light intensity, which serves as the primary independent variable under the experimental conditions of this study. Therefore, both the autogenic regulation term and the environmental disturbance term can be expressed as functions of light intensity. The function ${\phi }_{i}\left(l\right)$ represents the optimal fitting function of phenotype *i*, which is constructed based on the classical growth equation with logistic features, comprising of exponential growth, linear growth and asymptotic growth [49]. It can be mathematically expressed as Eq. (3), where the parameters *K* all represent the upper asymptotic values of autogenic regulation terms. In the Gompertz, Logistic, Mitscherlich, and Bertalanffy equations, *a* is a parameter related to the initial value, *b* denotes the inherent growth rate. In the Richards equation, *a* determines the size of the growth factor when *l* = 0, b is related to the growth rate, and *m* determines the shape of the curve and the position of the inflection point. In the Korf equation, *a* is related to the relative change rate of autogenic regulation terms, *b* determines the position of the turning point. In the Weibull equation, *a* is the scale parameter, *b* is the shape parameter, and the equation has an inflection point when *b* > 0. The function ${\omega }_{i}\left(l\right)$ is a disturbance function designed to evaluate the impact of the light environment according to an allometric scaling law. It is mathematically described by Eq. (4), where ${c}_{i}$ represents the disturbance rate and ${d}_{i}$ represents the disturbance scale coefficient [50]. The term ${\epsilon }_{ij}$ represents the disturbance term of the population structure for the *j*th population corresponding to phenotype *i* of the natural samples. It accounts for the variation in phenotype among different populations and serves to correct for such differences.

3 $$\varphi_i(l)=optim\;\left\{\begin{array}{l}Ke^{-ae^{-bl}}\\\frac k{1+ae^{-bl}}\\K\;\left(1-ae^{-bl}\right)\\K\;\left(1-ae^{-bl}\right)^3\\K\;\left(1-ae^{-bl}\right)^\frac1{1-m}\\\begin{array}{l}Ke^{-\frac a{l^b}}\\K\;\left(1-e^{-\left(\frac1a\right)^{b}}\right)\end{array}\end{array}\left.\begin{array}{l}(Gompertz)\\(Logistic)\\(Mitscherlich)\\(Bertealanffy)\\(Richards)\\\begin{array}{l}(Korf)\\\;(Weibull)\end{array}\end{array}\right\}\right.$$

4 $${\omega }_{i}\left(e\right)={c}_{i}{l}^{{d}_{i}}$$

### Modeling framework of QTL mapping

Let *n* denote the number of samples in the mapping population of *Populus simonii*, and all samples were genotyped by genome-wide SNPs and phenotyped across a range of light intensities, denoted as *L*. Using ${y}_{ik}=({y}_{ik}\left(1\right),\dots ,{y}_{ik}\left(L\right)$, where *i* represents ETR, qP, or qN, and *k* represents the *Populus simonii* sample (*k* = 1, 2,…, *n*), represents the photosynthetic phenotypic values measured at light intensity 1,…, *L*. Under the assumption that the phenotypes approximately follow normal distributions, the mixture likelihood model for phenotypic trait *i* at a given QTL is expressed as:

5 $$L\left({y}_{i}\right)={\prod }_{g=1}^{G}{\prod }_{k=1}^{{n}_{g}}{f}_{ig}\left({y}_{ik}\right)$$

where ${f}_{ig}\left({y}_{ik}\right)$ is the probability density function of the *L*-dimensional normal distribution of trait *i* with genotype $g$ ($g$ = 1, …, *G*). ${n}_{g}$ is the number of samples carrying genotype $g$, satisfying $\sum _{g=1}^{G}{n}_{g}=n$. The mixed likelihood model is determined by the mean vector ${\mu }_{g}$ and the covariance matrix Σ. ${\mu }_{g}$ represents the average phenotype of all samples with genotype $g$ at the specified QTL of phenotype *i* under *L* light intensities, as fitted by Eq. (2). The covariance matrix for each phenotype can be established using the first-order autoregressive (AR(1)) model [51], taking into account the phenotypic standard deviation under the initial light intensity. The model assumes that the residual variance and covariance remain stationary across different light intensities, thus, two reduced parameters are employed to model the residual covariance matrix for specific light intensities. The parameters Θ, which consist of the mean and covariance matrix in the model, are estimated using the maximum likelihood method. The estimation process is conducted through the expectation maximization (EM) [52] algorithm combined with the Nelder‒Mead simplex optimization method [53]. The EM algorithm is an iterative optimization method used to address parameter estimation problems in statistical models involving latent variables. The Nelder-Mead algorithm is an iterative optimization algorithm for unconstrained optimization problems, employed for parameter tuning and estimation in computations. If a QTL significantly regulates and influences the dynamic response of the phenotype to light intensity, the genotypic parameter vector ${{\Theta }}_{g}$ should reject the following null hypothesis and accept the alternative hypothesis:

6 $$H_0:\Theta\equiv\Theta_g\;\mathrm{versus}\mathit\;H_1:\Theta_g\neq\Theta,\;\mathrm{for}\;g=1,\cdots,G$$

The statistic under the null hypothesis is ${L}_{0}=L\left({y}_{i};{\Theta }\right)$, the statistic under the alternative hypothesis is ${L}_{1}=L({y}_{i};{\varTheta }_{g})$, and the difference in degrees of freedom between ${L}_{1}$ and ${L}_{0}$ corresponds to the difference in the number of parameters between the two hypotheses. The likelihood ratio test statistic is constructed as follows:

7 $$LR=-2({log}{L}_{0}-{log}{L}_{1})$$

The *p*-value (a metric used in statistical hypothesis testing to assess the consistency of the observed data with the null hypothesis) for different QTL loci can be calculated using the chi-square distribution, providing the basis for testing whether there are differences in trait development among genotypes. Under the assumption of non-normal distribution of phenotypes, the multivariate *t*-distribution mapping framework is a rational approach for QTL mapping [54], characterized by robust and powerful features, avoiding false positive issues caused by deviations from normality.

### Genetic network construction

Suppose ${P}_{i}$ significant QTLs are detected, which regulate the dynamic response of photosynthetic phenotypes to light intensity. Based on the similar genetic effects of these sites on phenotypes, there may be interactions among them that shape the response of photosynthetic phenotypes to changes in light intensity, ultimately facilitating improved plant adaptation. According to evolutionary game theory [55], any strategy employed by an individual to maximize its gains is influenced by the strategies of other individuals, leading to dynamic decision-making and an ongoing evolutionary process. This is a mathematical model that applies the concepts of game theory to study the evolutionary behavior of individuals (individual SNPs) within a population. It integrates ideas from evolutionary biology and game theory, aiming to understand how individual SNPs across the entire genome select strategies through genetic and adaptive learning to gain an advantage in competition. Based on the differential equation model, the genetic effect of each QTL on the phenotype was decomposed into its direct effect and the indirect effects mediated by other QTLs through various pathways. Let ${g}_{\text{i}p}=({g}_{\text{i}\text{p}}\left(1\right),\cdots ,{g}_{\text{i}\text{p}}\left(L\right))$ denote the vectors representing the overall genetic effects on trait *i* of QTL *p*. The model is expressed as follows:

8 $$\frac{d{g}_{\text{i}p}}{dl}={F}_{ip}\left({g}_{\text{i}p};{{\Phi }}_{ip}\right)+\sum\nolimits _{{p}^{{\prime }}=1,{p}^{{\prime }}\ne p}^{{\text{P}}_{i}}{F}_{ip\leftarrow {ip}^{{\prime }}}\left({g}_{\text{i}{p}^{{\prime }}};{{\Phi }}_{ip\leftarrow {ip}^{{\prime }}}\right),p=1,\cdots ,{P}_{i}$$

where the rate of change of the genetic effect of QTL *p* on trait *i* in response to light intensity is decomposed into two components: the independent expression component, $F_{ip}(\cdot)$, specified by parameters ${{\Phi }}_{ip}$, and the dependent expression component, ${F}_{p\leftarrow {p}^{{\prime }}}(\cdot )$, specified by parameters ${{\Phi }}_{ip\leftarrow {ip}^{{\prime }}}$. According to the equation mentioned above, for any QTL *p* as a response, there are (${P}_{i}$ – 1) predictors, leading to high complexity that contradicts the sparse network theory. This complexity is not conducive to the stability of the network structure [53, 55, 56]. Thus, we employed variable selection based on adaptive least absolute shrinkage and selection operator (LASSO) to identify ${d}_{ip} ({d}_{ip}\ll {P}_{i})$ nonzero predictors, which represent the most significant QTLs interacting with QTL *p*. We computed the reciprocal set of absolute values of the coefficients for the optimal ridge regression model, providing individual penalization weights to the coefficients of each variable in the realization of the model [57–59]. The application of adaptive weights with oracle properties ensures robust variable selection results [60, 61]. By replacing ${P}_{i}$ by ${d}_{ip}$, Eq. (8) is simplified as:

9 $$\frac{d{g}_{\text{i}p}}{dl}={F}_{ip}\left({g}_{\text{i}p};{{\Phi }}_{ip}\right)+\sum\nolimits _{{p}^{{\prime }}=1,{p}^{{\prime }}\ne p}^{{\text{d}}_{ip}}{F}_{ip\leftarrow {ip}^{{\prime }}}\left({g}_{\text{i}{p}^{{\prime }}};{{\Phi }}_{ip\leftarrow {ip}^{{\prime }}}\right),p=1,\cdots ,{\text{P}}_{i}$$

Based on the least squares method, we utilize the nonparametric Legendre Orthogonal Polynomial (LOP) to obtain optimal coefficients for LOP, achieving a smooth estimation of genetic effects and solve the model using the least square method and the fourth-order Runge‒Kutta algorithm (an iterative method for numerically solving ordinary differential equations), which constructs weighted, directed, and signed networks.

### Supplementary Information

**Additional file 1:** **S Fig. 1.** Histograms of gradient light intensity and average photosynthetic phenotype (ETR, qP, and qN), along with the *p*-value from the Kolmogorov-Smirnov test. **S Fig. 2. **Manhattan plots of photosynthetic phenotypes (ETR, qP, and qN) under different light intensities based on static mapping. The red line is the threshold of significance level of 0.05. **S Fig. 3. **Gene function and number of candidate genes belonging to significant QTLs regulating ETR in *Populus trichocarpa*. **S Fig. 4. **Gene function and number of candidate genes belonging to significant QTL regulating qP in *Populus trichocarpa*. **S Fig. 5. **Gene function and number of candidate genes belonging to significant QTLs regulating qP in *Populus trichocarpa*. **S Fig. 6. **Genetic effect curves and decomposition of networks. Overall genetic effects (blue line) are decomposed into independent effects (red line) and dependent effects (green line) due to regulation by other SNPs.

**Additional file 2: S Table 1.** Fitting parameters and evaluation information for average phenotypic fitting. **S Table 2.** Estimation of perturbation parameters ε. **S Table 3. **QTL information within candidate genes that significantly regulate ETR. **S Table 4. **QTL information within candidate genes that significantly regulate qP. S Table 4. QTL information within candidate genes that significantly regulate qP. **S Table 5. **QTL information within candidate genes that significantly regulate qN.

## Data Availability

The computer code and data used in this study are available at https://github.com/HuiyingGong1102/HDEE or upon request directly from the corresponding author.

## Abbreviations

ETR: Electron transfer rates
qP: Photochemical quenching
qN: Nonphotochemical quenching
HDEE: High-dimensional equations with environmental regulators
QTL: Quantitative trait locus
SNP: Single nucleotide polymorphism
GO: Gene ontology
ATP: Adenosine Triphosphate
Pfam: Protein FAMilies database
PSII: Photosystem II
DNA: Deoxyribonucleic Acid
CTAB: Cetyltrimethylammonium Bromide
SAMtools: Sequence Alignment/Map tools
GATK4: Genome Analysis Toolkit 4.0
QD: Variant Quality by Depth
MQ: Mapping Quality
FS: Fisher Strand Bias
SOR: Strand Odds Ratio
MQRankSum: Mapping Quality Rank Sum
ReadPosRankSum: Read Position Rank Sum
vcftools: Variant Call Format tools
MAF: Minor allele frequency
AR(1): First-order autoregressive
EM: Expectation maximization
LASSO: Least absolute shrinkage and selection operator
LOP: Legendre orthogonal polynomial
RMSE: Root mean squared error
AIC: Akaike information criterion
BIC: Bayesian information criterion
HQ: Hannan-Quinn criterion

## References

[1] Schreier M, Curvat L, Giordano F, Steier L, Abate A, Zakeeruddin SM (2015). Efficient photosynthesis of carbon monoxide from CO2 using perovskite photovoltaics. Nat Commun.

[2] Flood PJ, Harbinson J, Aarts MGM (2011). Natural genetic variation in plant photosynthesis. Trends Plant Sci.

[3] Moore KA, Altus S, Tay JW, Meehl JB, Johnson EB, Bortz DM, Cameron JC (2020). Mechanical regulation of photosynthesis in cyanobacteria. Nat Microbiol.

[4] Stiller JW, Schreiber J, Yue J, Guo H, Ding Q, Huang J (2014). The evolution of photosynthesis in chromist algae through serial endosymbioses. Nat Commun.

[5] Yang Q, Blanco NE, Hermida-Carrera C, Lehotai N, Hurry V, Strand Å (2020). Two dominant boreal conifers use contrasting mechanisms to reactivate photosynthesis in the spring. Nat Commun.

[6] Mascoli V, Bersanini L, Croce R (2020). Far-red absorption and light-use efficiency trade-offs in chlorophyll f photosynthesis. Nat Plants.

[7] Porcar-Castell A, Malenovský Z, Magney T, van Wittenberghe S, Fernández-Marín B, Maignan F (2021). Chlorophyll a fluorescence illuminates a path connecting plant molecular biology to earth-system science. Nat Plants.

[8] Wang Y, Wang X, Sun S, Jin C, Su J, Wei J (2022). GWAS, MWAS and mGWAS provide insights into precision agriculture based on genotype-dependent microbial effects in foxtail millet. Nat Commun.

[9] Sun X, Xiong H, Jiang C, Zhang D, Yang Z, Huang Y (2022). Natural variation of DROT1 confers drought adaptation in upland rice. Nat Commun.

[10] Peremarti A, Marè C, Aprile A, Roncaglia E, Cattivelli L, Villegas D, Royo C (2014). Transcriptomic and proteomic analyses of a pale-green durum wheat mutant shows variations in photosystem components and metabolic deficiencies under drought stress. BMC Genomics.

[11] Feil R, Fraga MF (2012). Epigenetics and the environment: emerging patterns and implications. Nat Rev Genet.

[12] López-Maury L, Marguerat S, Bähler J (2008). Tuning gene expression to changing environments: from rapid responses to evolutionary adaptation. Nat Rev Genet.

[13] Ahmadi N, Barry MB, Frouin J, de Navascués M, Toure MA (2023). Genome scan of rice landrace populations collected across time revealed climate changes’ selective footprints in the genes network regulating flowering time. Rice.

[14] Muhammad I, Shalmani A, Ali M, Yang Q-H, Ahmad H, Li FB (2021). Mechanisms regulating the dynamics of photosynthesis under abiotic stresses. Front Plant Sci.

[15] de Miguel M, Cabezas J-A, María N, de, Sánchez-Gómez D, Guevara M-Á, Vélez M-D (2014). Genetic control of functional traits related to photosynthesis and water use efficiency in Pinus pinaster Ait. Drought response: integration of genome annotation, allele association and QTL detection for candidate gene identification. BMC Genomics.

[16] Jiang L, Liu J, Zhu X, Ye M, Sun L, Lacaze X, Wu R (2015). 2HiGWAS: a unifying high-dimensional platform to infer the global genetic architecture of trait development. Brief Bioinform.

[17] Li Y, Wu R (2010). Functional mapping of growth and development. Biol Rev.

[18] Ma C-X, Casella G, Wu R (2002). Functional mapping of quantitative trait loci underlying the character process: a theoretical Framework. Genetics.

[19] Wu R, Lin M (2006). Functional mapping — how to map and study the genetic architecture of dynamic complex traits. Nat Rev Genet.

[20] Wu R, Jiang L (2021). Recovering dynamic networks in big static datasets. Phys Rep.

[21] Winsor CP (1932). The gompertz curve as a growth curve. Proc Nat Acad Sci.

[22] Verhulst PF. Notice sur la loi que la population suit dans son accroissement. Correspondance mathématique et physique publiée par A. 1838. p. 113–21.

[23] Xiao Y, Huang G, You X, Zhu Q, Wang W, Kuang T (2021). Structural insights into cyanobacterial photosystem II intermediates associated with Psb28 and Tsl0063. Nat Plants.

[24] Hohmann-Marriott MF, Blankenship RE (2011). Evolution of photosynthesis. Annu Rev Plant Biol.

[25] Sánchez-Baracaldo P, Cardona T (2020). On the origin of oxygenic photosynthesis and cyanobacteria. New Phytol.

[26] Vinyard DJ, Ananyev GM, Charles Dismukes G (2013). Photosystem II: The reaction center of oxygenic photosynthesis. Annu Rev Biochem.

[27] Zabret J, Bohn S, Schuller SK, Arnolds O, Möller M, Meier-Credo J (2021). Structural insights into photosystem II assembly. Nat Plants.

[28] Barber J (2003). Photosystem II: the engine of life. Q Rev Biophys.

[29] Hildner R, Brinks D, Nieder JB, Cogdell RJ, van Hulst NF (2013). Quantum coherent energy transfer over varying pathways in single light-harvesting complexes. Science.

[30] Mohseni M, Rebentrost P, Lloyd S, Aspuru-Guzik A (2008). Environment-assisted quantum walks in photosynthetic energy transfer. J Chem Phys.

[31] Shen J-R (2015). The structure of photosystem II and the mechanism of water oxidation in photosynthesis. Annu Rev Plant Biol.

[32] Heyneke E, Fernie AR (2018). Metabolic regulation of photosynthesis. Biochem Soc Trans.

[33] Yan X, Qu C, LI J, Chen L, Liu L. QTL analysis of leaf photosynthesis rate and related physiological traits in Brassica napus. 2095–3119. 2015;14:1261–8. 10.1016/S2095-3119(14)60958-8.

[34] Hervé D, Fabre F, Berrios EF, Leroux N, Chaarani GA, Planchon C (2001). QTL analysis of photosynthesis and water status traits in sunflower (Helianthus annuus L.) under greenhouse conditions. J Exp Bot.

[35] Takai T, Kondo M, Yano M, Yamamoto T (2010). A quantitative trait locus for chlorophyll content and its association with leaf photosynthesis in rice. Rice.

[36] Teng S, Qian Q, Zeng D, Kunihiro Y, Fujimoto K, Huang D, Zhu L (2004). QTL analysis of leaf photosynthetic rate and related physiological traits in rice (Oryza sativa L). Euphytica.

[37] Xu Y-F, Li S-S, Li L-H, Ma F-F, Fu X-Y, Shi Z-L (2017). QTL mapping for yield and photosynthetic related traits under different water regimes in wheat. Mol Breeding.

[38] Sun J, Xie D, Zhang E, Zheng H, Wang J, Liu H (2019). QTL mapping of photosynthetic-related traits in rice under salt and alkali stresses. Euphytica.

[39] Liu X, Fan Y, Mak M, Babla M, Holford P, Wang F (2017). QTLs for stomatal and photosynthetic traits related to salinity tolerance in barley. BMC Genomics.

[40] Ci D, Song Y, Du Q, Tian M, Han S, Zhang D (2016). Variation in genomic methylation in natural populations of Populus simonii is associated with leaf shape and photosynthetic traits. J Exp Bot.

[41] Xiao L, Liu X, Lu W, Chen P, Quan M, Si J (2020). Genetic dissection of the gene coexpression network underlying photosynthesis in Populus. Plant Biotechnol J.

[42] Solovieff N, Cotsapas C, Lee PH, Purcell SM, Smoller JW (2013). Pleiotropy in complex traits: challenges and strategies. Nat Rev Genet.

[43] Lawlor DW (1995). Photosynthesis, productivity and environment. J Exp Bot.

[44] Dogutan DK, Nocera DG (2019). Artificial Photosynthesis at efficiencies greatly exceeding that of natural photosynthesis. Acc Chem Res.

[45] Kaiser E, Morales A, Harbinson J, Kromdijk J, Heuvelink E, Marcelis LFM (2015). Dynamic photosynthesis in different environmental conditions. J Exp Bot.

[46] Farooq S, Chmeliov J, Wientjes E, Koehorst R, Bader A, Valkunas L (2018). Dynamic feedback of the photosystem II reaction centre on photoprotection in plants. Nat Plants.

[47] Liu P, Bu C, Chen P, El-Kassaby YA, Zhang D, Song Y (2023). Enhanced genome-wide association reveals the role of YABBY11-NGATHA-LIKE1 in leaf serration development of Populus. Plant Physiol.

[48] Niklas KJ, Marler TE (2007). Carica papaya (Caricaceae): a case study into the effects of domestication on plant vegetative growth and reproduction. Am J Bot.

[49] West GB, Brown JH, Enquist BJ (2001). A general model for ontogenetic growth. Nature.

[50] Voje KL, Hansen TF, Egset CK, Bolstad GH, Pélabon C (2014). Allometric constraints and the evolution of Allometry. Evol.

[51] Zhao W, Hou W, Littell RC, Wu R (2005). Structured antedependence models for functional mapping of multiple longitudinal traits. Stat Appl Genet Mol Biol.

[52] Dempster AP, Laird NM, Rubin DB (1977). Maximum likelihood from incomplete data via the EM algorithm. J R Stat Soc B (Methodological).

[53] Zhao W, Wu R, Ma C-X, Casella G (2004). A fast algorithm for functional mapping of complex traits. Genetics.

[54] Wu C, Li G, Zhu J, Cui Y (2011). Functional mapping of dynamic traits with robust t-distribution. PLoS One.

[55] Smith JM, Price GR (1973). The logic of animal conflict. Nature.

[56] May RM (1972). Will a large complex system be stable?. Nature.

[57] Zou H (2006). The adaptive Lasso and its oracle properties. J Am Stat Assoc.

[58] Huang J, Ma S, Zhang CH (2008). Adaptive Lasso for sparse high-dimensional regression models. Stat Sin.

[59] Feldman CH, Yoshida K, Xu C, Frits ML, Shadick NA, Weinblatt ME (2019). Supplementing claims data with electronic medical records to improve estimation and classification of rheumatoid arthritis disease activity: a machine learning approach. ACR Open Rheumatol.

[60] Wu C, Ma S (2015). A selective review of robust variable selection with applications in bioinformatics. Brief Bioinform.

[61] Lu W, Goldberg Y, Fine JP (2012). On the robustness of the adaptive lasso to model misspecification. Biometrika.

