# Supplementary material for: Computational dissection of genetic variation modulating the response of multiple photosynthetic phenotypes to the light environment
Source: BMC Genomics. 2024 Jan 20;25:81. doi: 10.1186/s12864-024-09968-8 (PMC10799405; doi:10.1186/s12864-024-09968-8)
Supplement: Supplementary file 1 — Additional file 1: S Fig. 1. Histograms of gradient light intensity and average photosynthetic phenotype (ETR, qP, and qN), along with the p-value from the Kolmogorov-Smirnov test. S Fig. 2. Manhattan plots of photosynthetic phenotypes (ETR, qP, and qN) under different light intensities based on static mapping. The red line is the threshold of significance level of 0.05. S Fig. 3. Gene function and number of candidate genes belonging to significant QTLs regulating ETR in Populus trichocarpa. S Fig. 4. Gene function and number of candidate genes belonging to significant QTL regulating qP in Populus trichocarpa. S Fig. 5. Gene function and number of candidate genes belonging to significant QTLs regulating qP in Populus trichocarpa. S Fig. 6. Genetic effect curves and decomposition of networks. Overall genetic effects (blue line) are decomposed into independent effects (red line) and dependent effects (green line) due to regulation by other SNPs. [file 12864_2024_9968_MOESM1_ESM.docx]

**S Fig. 1.** Histograms of gradient light intensity and average photosynthetic phenotype (ETR, qP, and qN), along with the *p*-value from the Kolmogorov-Smirnov test.


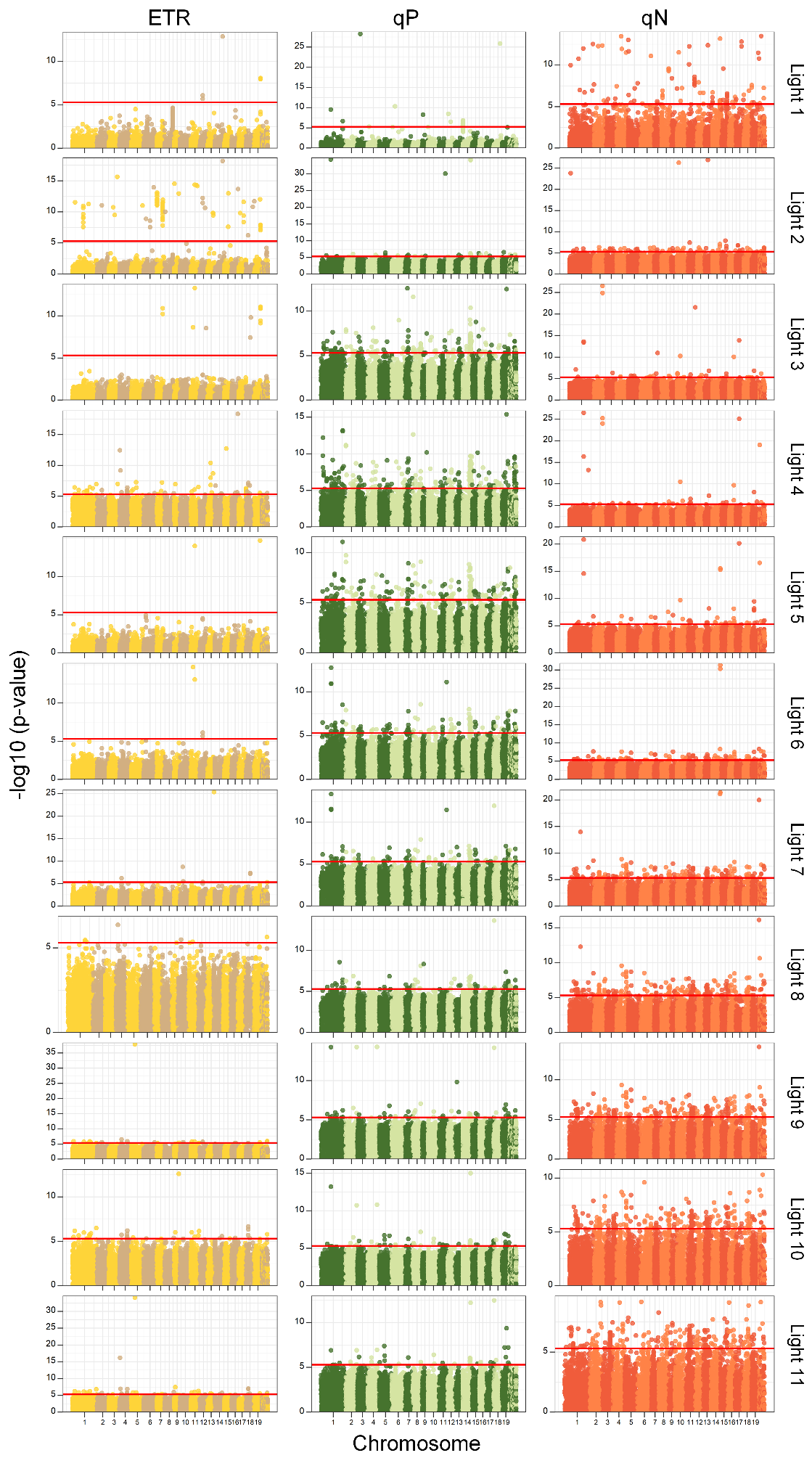


**S Fig. 2.** Manhattan plots of photosynthetic phenotypes (ETR, qP, and qN) under different light intensities based on static mapping. The red line is the threshold of significance level of 0.05.


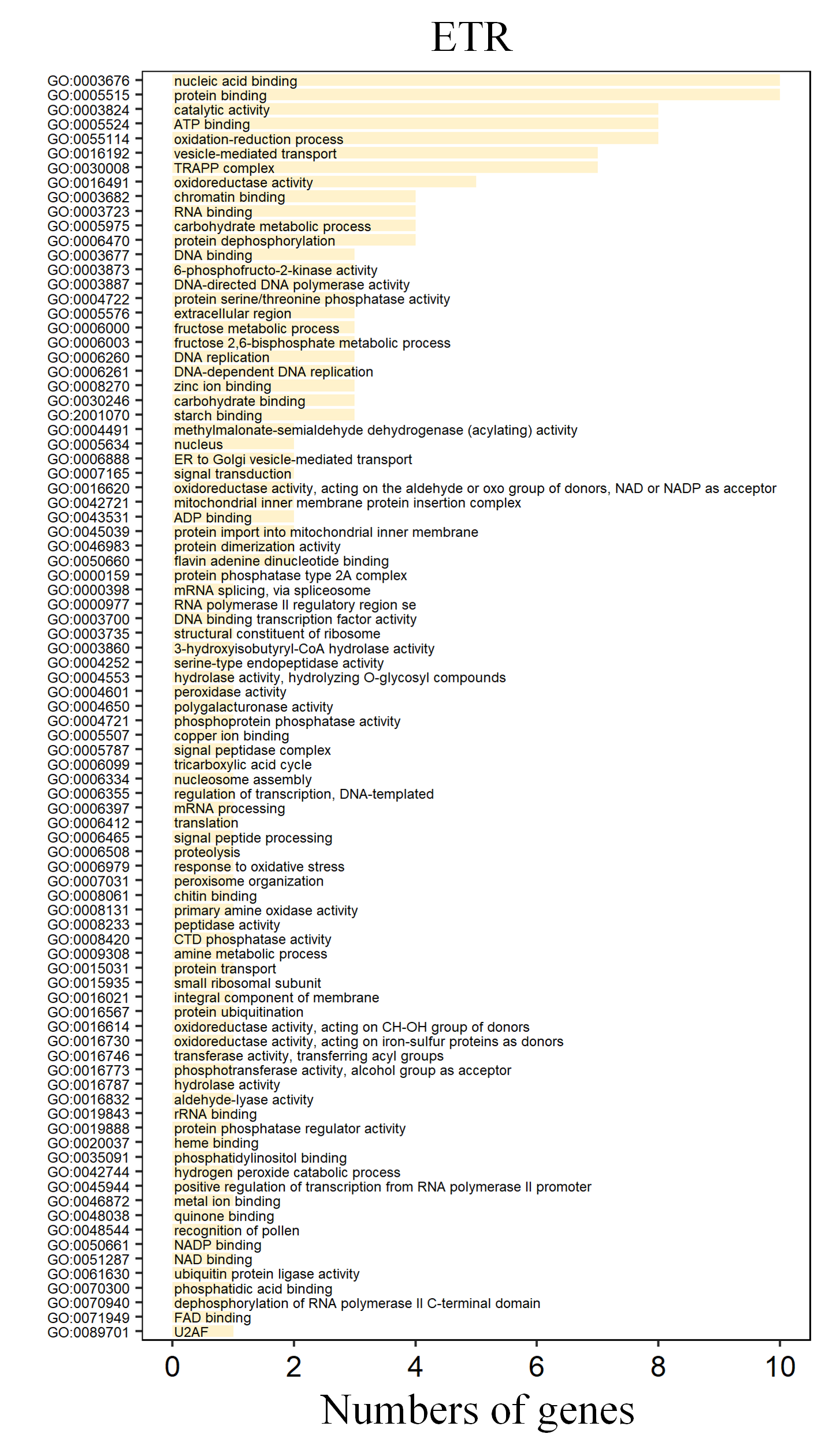


**S Fig. 3.** Gene function and number of candidate genes belonging to significant QTLs regulating ETR in *Populus trichocarpa*.


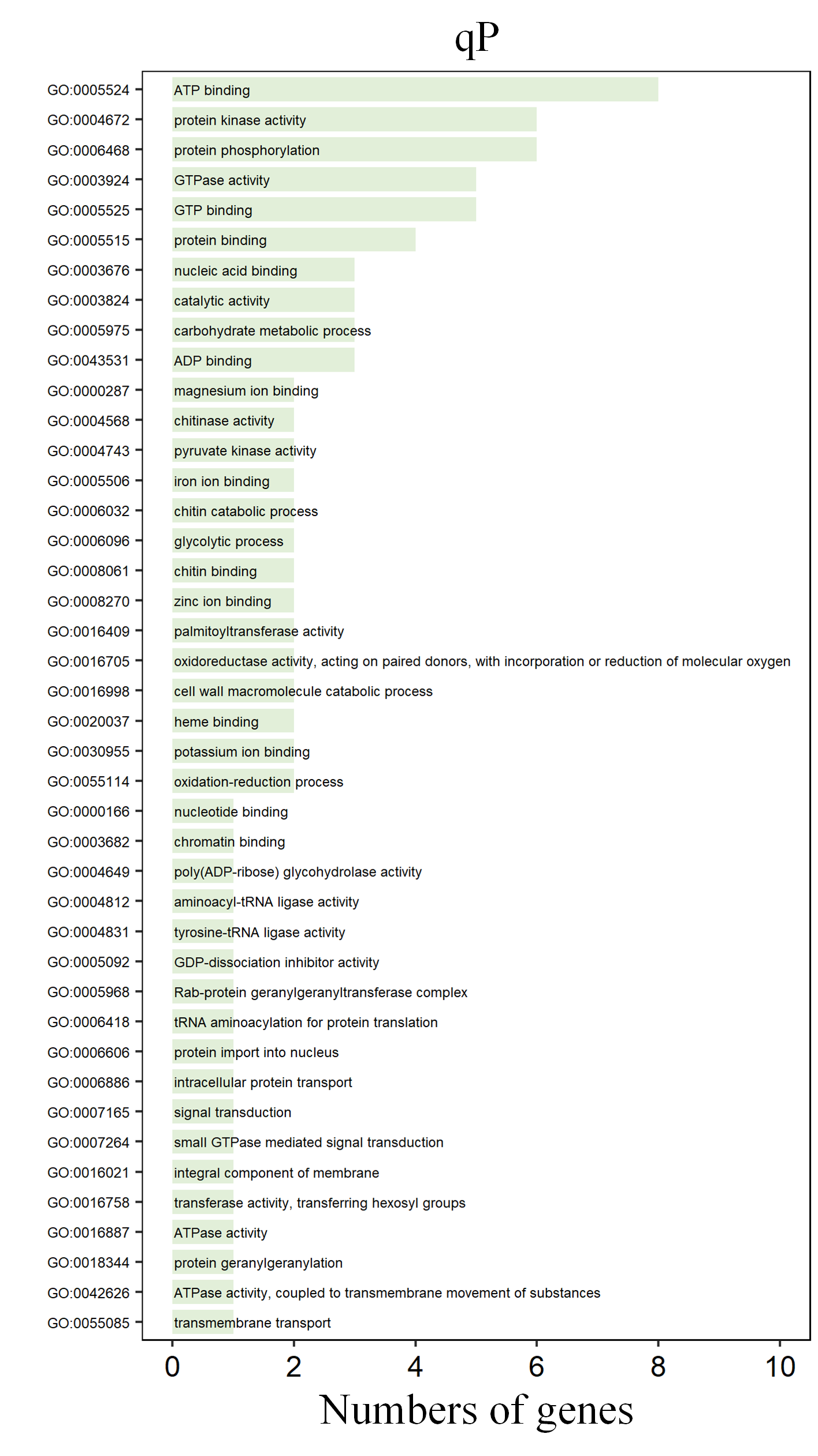


**S Fig. 4.** Gene function and number of candidate genes belonging to significant QTL regulating qP in *Populus trichocarpa*.


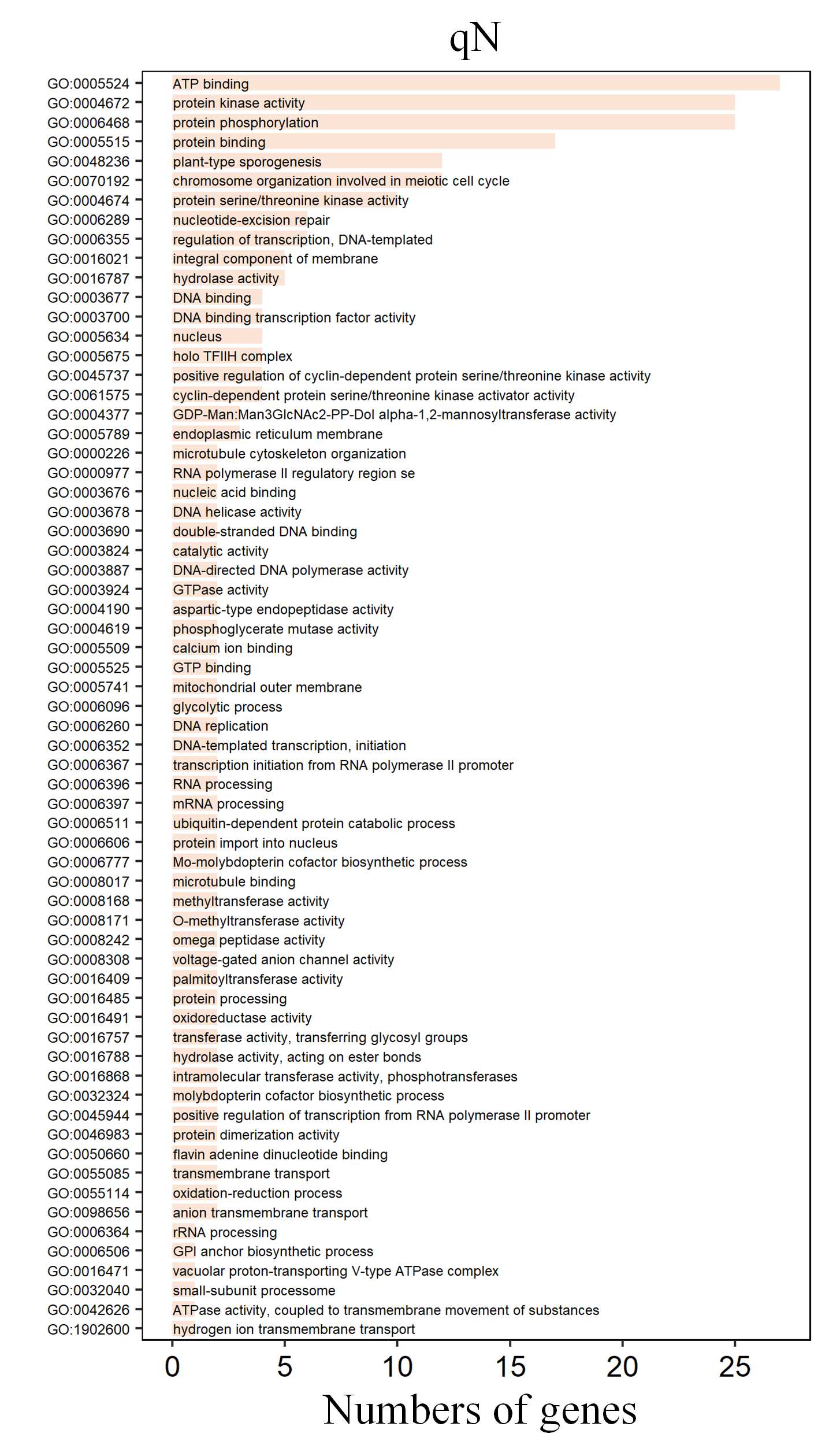


**S Fig. 5.** Gene function and number of candidate genes belonging to significant QTLs regulating qP in *Populus trichocarpa*.


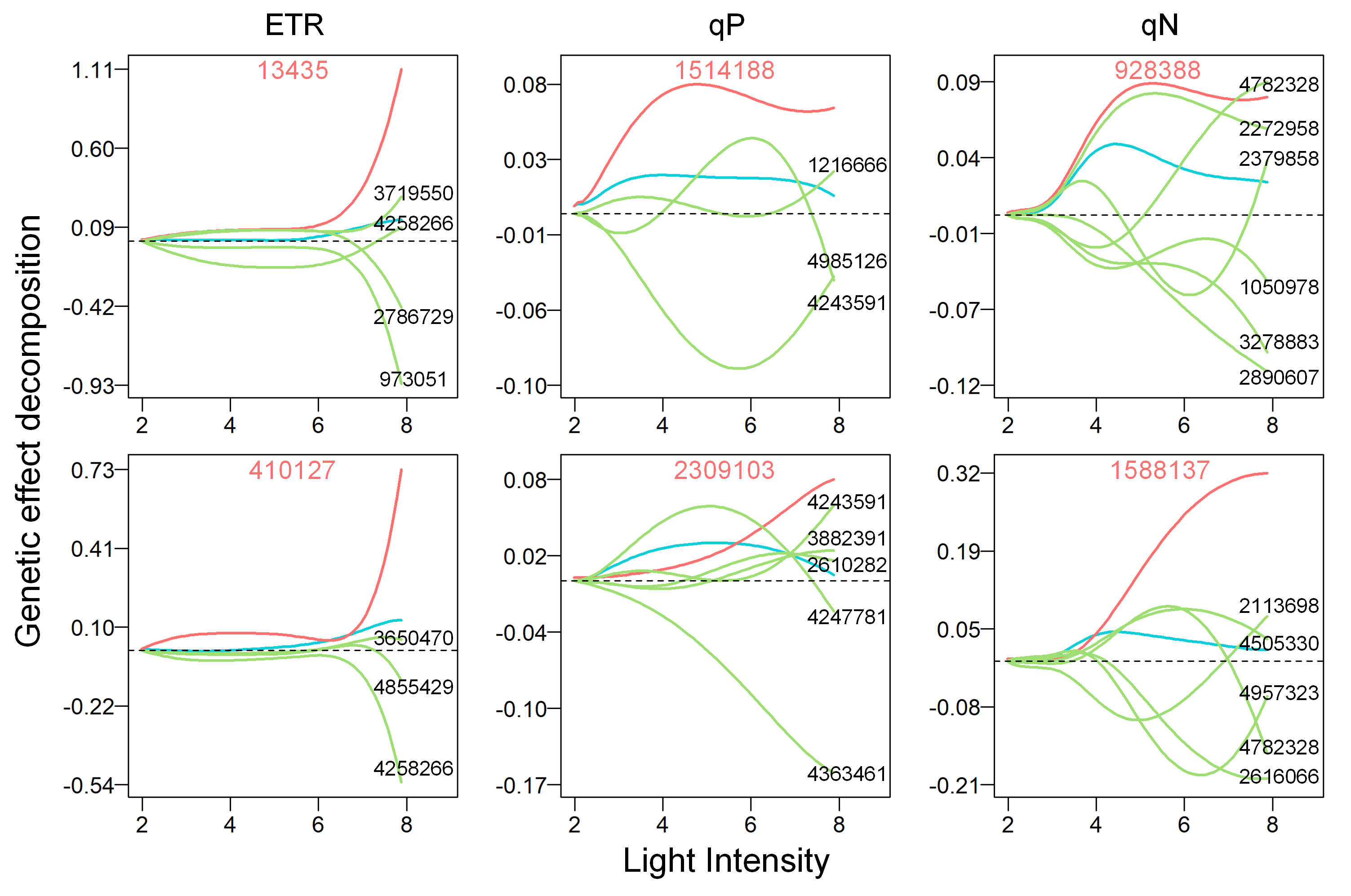


**S Fig. 6.** Genetic effect curves and decomposition of networks. Overall genetic effects (blue line) are decomposed into independent effects (red line) and dependent effects (green line) due to regulation by other SNPs.
