# Supplementary material for: Computational dissection of genetic variation modulating the response of multiple photosynthetic phenotypes to the light environment
Source: BMC Genomics. 2024 Jan 20;25:81. doi: 10.1186/s12864-024-09968-8 (PMC10799405; doi:10.1186/s12864-024-09968-8)
Supplement: Supplementary file 2 — Additional file 2: S Table 1. Fitting parameters and evaluation information for average phenotypic fitting. S Table 2. Estimation of perturbation parameters ε. S Table 3. QTL information within candidate genes that significantly regulate ETR. S Table 4. QTL information within candidate genes that significantly regulate qP. S Table 4. QTL information within candidate genes that significantly regulate qP. S Table 5. QTL information within candidate genes that significantly regulate qN. [file 12864_2024_9968_MOESM2_ESM.docx]

**S Table 1.** Fitting parameters and evaluation information for average phenotypic fitting.

|  | ETR (Electron transfer rate in PSⅡ) | | | | | | | | | | |
| --- | --- | --- | --- | --- | --- | --- | --- | --- | --- | --- | --- |
|  | $K$ | $a$ | $b$ | $m$ | $c$ | $d$ | $RMSE$ | $R^{2}$ | $-AIC$ | $-BIC$ | $-HQ$ |
| HDEE | 1.038 | 3109.000 | 1.339 | - | 0.009 | 1.328 | 9.063$\times{10}^{-5}$ | 9.999$\times{10}^{-1}$ | 92.396 | 78.417 | 98.023 |
| LE | 1.244 | 900.000 | 1.132 | - | - | - | 1.438$\times{10}^{-4}$ | 9.990$\times{10}^{-1}$ | 91.316 | 82.928 | 94.692 |
| ME | 10.535 | 1.065 | 0.021 | - | - | - | 1.379$\times{10}^{-2}$ | 9.013$\times{10}^{-1}$ | 41.124 | 32.737 | 44.500 |
| RE | 30.517 | 1.021 | 0.086 | 0.917 | - | - | 1.281$\times{10}^{-2}$ | 9.083$\times{10}^{-1}$ | 39.930 | 28.747 | 45.306 |
| KE | 3.402 | 34.671 | 1.678 | - | - | - | 1.044$\times{10}^{-3}$ | 9.925$\times{10}^{-1}$ | 69.513 | 61.126 | 72.889 |
| GE | 1.541 | 26.739 | 0.565 | - | - | - | 5.910$\times{10}^{-4}$ | 9.958$\times{10}^{-1}$ | 75.770 | 67.383 | 79.146 |
| BE | 2.167 | 2.171 | 0.308 | - | - | - | 1.019$\times{10}^{-3}$ | 9.927$\times{10}^{-1}$ | 69.776 | 61.389 | 73.152 |
| WE | 1.161 | 6.329 | 5.08 | - | - | - | 1.756$\times{10}^{-4}$ | 9.987$\times{10}^{-1}$ | 89.118 | 80.731 | 92.494 |
|  | qP (Photochemical quenching) | | | | | | | | | | |
|  | $K$ | $a$ | $b$ | $m$ | $c$ | $d$ | $RMSE$ | $R^{2}$ | $-AIC$ | $-BIC$ | $-HQ$ |
| HDEE | 0.805 | 0.040 | -0.399 | - | 0.273 | -0.422 | 4.935$\times{10}^{-4}$ | 9.904$\times{10}^{-1}$ | 73.754 | 59.775 | 79.381 |
| LE | 0.898 | 0.002 | -0.917 | - | - | - | 1.010$\times{10}^{-3}$ | 9.803$\times{10}^{-1}$ | 69.880 | 61.493 | 73.256 |
| ME | 1.063 | 0.067 | -0.320 | - | - | - | 5.318$\times{10}^{-4}$ | 9.896$\times{10}^{-1}$ | 76.932 | 68.545 | 80.309 |
| RE | 0.028 | -53.329 | 0.430 | 0.570 | - | - | 1.026$\times{10}^{-2}$ | 7.994$\times{10}^{-1}$ | 42.369 | 31.186 | 47.746 |
| KE | 1.039 | 0.008 | -2.448 | - | - | - | 3.079$\times{10}^{-3}$ | 9.398$\times{10}^{-1}$ | 57.613 | 49.230 | 60.990 |
| GE | 0.888 | 0.005 | -0.743 | - | - | - | 8.524$\times{10}^{-4}$ | 9.833$\times{10}^{-1}$ | 71.742 | 63.354 | 75.118 |
| BE | 1.040 | 0.014 | -0.431 | - | - | - | 7.207$\times{10}^{-4}$ | 9.859$\times{10}^{-1}$ | 73.588 | 65.200 | 76.964 |
| WE | 0.814 | 6.241 | -4.784 | - | - | - | 2.588$\times{10}^{-3}$ | 9.494$\times{10}^{-1}$ | 59.525 | 51.137 | 62.901 |
|  | qN (Nonphotochemical quenching) | | | | | | | | | | |
|  | $K$ | $a$ | $b$ | $m$ | $c$ | $d$ | $RMSE$ | $R^{2}$ | $-AIC$ | $-BIC$ | $-HQ$ |
| HDEE | 0.874 | 168.817 | 3.407 | - | 0.184 | -4.160 | 1.109$\times{10}^{-5}$ | 9.998$\times{10}^{-1}$ | 115.503 | 101.524 | 121.130 |
| LE | 0.753 | 360.000 | 1.225 | - | - | - | 2.091$\times{10}^{-4}$ | 9.963$\times{10}^{-1}$ | 87.202 | 78.815 | 90.578 |
| ME | 2.957 | 1.128 | 0.055 | - | - | - | 1.957$\times{10}^{-3}$ | 9.650$\times{10}^{-1}$ | 62.600 | 54.212 | 65.976 |
| RE | 0.496 | 3.793 | 2.040 | -1.920 | - | - | 5.594$\times{10}^{-2}$ | 2.235$\times{10}^{-1}$ | 23.718 | 12.535 | 29.094 |
| KE | 0.874 | 167.749 | 3.405 | - | - | - | 2.175$\times{10}^{-5}$ | 9.996$\times{10}^{-1}$ | 112.093 | 103.705 | 115.469 |
| GE | 0.789 | 38.000 | 0.828 | - | - | - | 5.743$\times{10}^{-5}$ | 9.990$\times{10}^{-1}$ | 101.413 | 93.026 | 104.790 |
| BE | 0.849 | 4.031 | 0.597 | - | - | - | 1.393$\times{10}^{-4}$ | 9.975$\times{10}^{-1}$ | 91.665 | 83.278 | 95.042 |
| WE | 0.741 | 5.244 | 3.996 | - | - | - | 2.586$\times{10}^{-4}$ | 9.954$\times{10}^{-1}$ | 84.863 | 76.476 | 88.239 |

**S Table 2.** Estimation of perturbation parameters ε.

| ε  Population | ε (ETR) | ε (qP) | ε (qN) |
| --- | --- | --- | --- |
| group1 | -0.052  (0.162) | 0.294  (0.725) | 0.333  (0.857) |
| group 2 | -0.206  (0.298) | -0.004  (0.046) | 0.282  (0.555) |
| group 3 | -0.288  (0.320) | 0.056  (0.232) | 0.374  (0.818) |
| group 4 | -0.110  (0.194) | 0.095  (0.184) | 0.330  (1.047) |
| group 5 | -0.228  (0.259) | 0.119  (0.202) | 0.123  (1.138) |
| group 6 | -0.285  (0.305) | 0.103  (0.190) | 0.050  (1.370) |
| p-value | 0.089 | 0.315 | 0.944 |

Average parameter values are calculated for different populations of phenotypes in photosystem II (ETR, qP, and qN) in Formula (2). The parameters in parentheses are the standard deviations of the parameters. The p-values of the ANOVA of perturbation parameters for different groups of population in the last row are greater than 0.05, indicating that the differences in parameter values between populations are not significant.

**S Table 3.** QTL information within candidate genes that significantly regulate ETR.

| SNP | Chr | Position | Gene | Function in *Populus trichocarpa* | Homologous gene in  *Arabidopsis thaliana* |
| --- | --- | --- | --- | --- | --- |
| 13434 | Chr01 | 875077 | Potri.001G012900 | - | - |
| 13435 | Chr01 | 875079 | Potri.001G012900 | - | - |
| 57586 | Chr01 | 4757386 | Potri.001G061500 | - | AT2G33490, AT3G26910, AT5G41100 |
| 142642 | Chr01 | 11980480 | Potri.001G147200 | GO:0005576  PF01357  PF03330 | AT3G45960, AT3G45970, AT4G17030, AT4G38400 |
| 142649 | Chr01 | 11980647 | Potri.001G147200 | GO:0005576  PF01357  PF03330 | AT3G45960, AT3G45970, AT4G17030, AT4G38400 |
| 142656 | Chr01 | 11981088 | Potri.001G147200 | GO:0005576 PF01357 PF03330 | AT3G45960, AT3G45970, AT4G17030, AT4G38400 |
| 150669 | Chr01 | 12617160 | Potri.001G153000 | GO:0003723 PF04774 PF09598 | AT4G16830, AT4G17520, AT5G47210 |
| 150702 | Chr01 | 12619076 | Potri.001G153000 | GO:0003723  PF04774  PF09598 | AT4G16830, AT4G17520, AT5G47210 |
| 368173 | Chr01 | 26374476 | Potri.001G253900 | PF13460 | AT4G31530 |
| 374167 | Chr01 | 26750605 | Potri.001G258500 | GO:0005515  PF01535  PF13041 | AT1G04840, AT1G06140, AT1G06143,  etc. (152) |
| 410101 | Chr01 | 28940000 | Potri.001G283100 | GO:0004491 GO:0016620 GO:0055114 GO:0016491 PF00171 | AT2G14170 |
| 410127 | Chr01 | 28940957 | Potri.001G283100 | GO:0004491 GO:0016620 GO:0055114 GO:0016491 PF00171 | AT2G14170 |
| 437153 | Chr01 | 30802691 | Potri.001G305100 | GO:0046983 PF00010 | AT1G49770 |
| 509975 | Chr01 | 35776571 | Potri.001G350900 | GO:0055114 GO:0016730 PF02943 | AT2G04700 |
| 554192 | Chr01 | 38287950 | Potri.001G368900 | PF05627 | AT2G04410, AT2G17660, AT3G48450, AT4G35655, AT5G40645, AT5G55850, AT5G63270 |
| 611167 | Chr01 | 41925799 | Potri.001G398300 | GO:0016491 | - |
| 701421 | Chr01 | 49780055 | Potri.001G463500 | GO:0050660 GO:0055114 GO:0071949 GO:0016491 PF01565 | AT1G01980, AT1G11770, AT1G26380,  etc. (27) |
| 768879 | Chr02 | 6964753 | Potri.002G096500 | PF15982 | AT1G34630 |
| 768888 | Chr02 | 6965892 | Potri.002G096500 | PF15982 | AT1G34630 |
| 768893 | Chr02 | 6966123 | Potri.002G096500 | PF15982 | AT1G34630 |
| 768919 | Chr02 | 6967527 | Potri.002G096500 | PF15982 | AT1G34630 |
| 813877 | Chr02 | 11439158 | Potri.002G151700 | GO:0003700 GO:0005634 GO:0006355 GO:0045944 GO:0000977 PF00319 | AT1G17310, AT1G22130, AT1G24260, AT1G26310, AT1G69120,  etc. (38) |
| 977004 | Chr02 | 23674048 | Potri.002G245200 | GO:0005515 PF01535 PF13041 | AT1G04840, AT1G06140, AT1G06143,  etc. (152) |
| 985979 | Chr02 | 24528718 | Potri.002G255900 | PF05368 | AT2G20360 |
| 988096 | Chr02 | 24815484 | Potri.002G259600 | GO:0003824 GO:0006099 GO:0005524 PF08442 PF00549 | AT2G20420 |
| 988096 | Chr02 | 24815484 | Potri.002G259700 | GO:0050660 GO:0016614 GO:0055114 PF05199 PF00732 | AT1G03990, AT3G23410, AT4G19380, AT4G28570 |
| 1003773 | Chr03 | 775233 | Potri.003G009650 | - | - |
| 1019855 | Chr03 | 2565139 | Potri.003G021400 | GO:0005634 GO:0006334 PF00956 | AT2G19480, AT3G13782, AT4G26110, AT5G56950 |
| 1104421 | Chr03 | 7886419 | Potri.003G053200 | GO:0035091 PF02194 PF08628 PF00787 | AT1G15240 |
| 1141698 | Chr03 | 10679369 | Potri.003G078800 | GO:0016567 GO:0061630 GO:0008270 GO:0003676 GO:0006397 PF13696 PF08783 | AT4G17410, AT5G47430 |
| 1224754 | Chr03 | 18979973 | Potri.003G183400 | GO:0006888 PF04628 | AT1G80500 |
| 1224756 | Chr03 | 18979993 | Potri.003G183400 | GO:0006888  PF04628 | AT1G80500 |
| 1281123 | Chr04 | 2154546 | Potri.004G029500 | GO:0070300 | AT1G11440, AT3G29075 |
| 1355883 | Chr04 | 8072139 | Potri.004G094500 | PF12874 | AT1G60640 |
| 1458209 | Chr04 | 15913043 | Potri.004G137401 | - | - |
| 1467363 | Chr04 | 16448664 | Potri.004G141100 | - | - |
| 1482822 | Chr04 | 17446510 | Potri.004G152600 | - | AT1G49330, AT2G16190 |
| 1827478 | Chr05 | 20425440 | Potri.005G186900 | PF01803 | AT1G43850 |
| 1921730 | Chr06 | 4323574 | Potri.006G059300 | GO:0042721 GO:0045039  PF02466 | AT2G42210 |
| 1921753 | Chr06 | 4326647 | Potri.006G059300 | GO:0042721 GO:0045039 PF02466 | AT2G42210 |
| 1968869 | Chr06 | 8326750 | Potri.006G107000 | GO:0042744 GO:0055114 GO:0004601 GO:0020037 GO:0006979 PF00141 | AT1G05240, AT1G05250, AT1G05260,  etc. (48) |
| 1979496 | Chr06 | 9158159 | Potri.006G116700 | GO:0089701 GO:0046872 GO:0000398 GO:0003676 GO:0003723 PF00642 PF00076 | AT1G27650, AT3G44785, AT5G42820 |
| 2122183 | Chr06 | 19999654 | Potri.006G185900 | GO:0070940 GO:0008420 | AT2G33540, AT5G58003 |
| 2160969 | Chr06 | 22465265 | Potri.006G209700 | GO:0003735 GO:0006412 GO:0015935 GO:0003723 GO:0019843 PF01479 PF00163 | AT5G15200, AT5G39850 |
| 2179621 | Chr06 | 24159984 | Potri.006G231000 | GO:0019888 GO:0000159 GO:0005515 PF00400 | AT1G17720, AT1G51690 |
| 2196986 | Chr06 | 25782270 | Potri.006G252300 | GO:0005524 PF03969 | AT2G25530 |
| 2407186 | Chr07 | 14011721 | Potri.007G121600 | GO:0007031 | AT3G21865 |
| 2425364 | Chr07 | 15488898 | Potri.007G145600 | - | - |
| 2425364 | Chr07 | 15488898 | Potri.007G145700 | - | AT2G31840, AT4G28590 |
| 2462397 | Chr08 | 4986315 | Potri.008G079900 | GO:0004252 GO:0005515 GO:0006508 PF13180 PF17815 PF13365 | AT1G65630, AT1G65640, AT5G36950, AT5G40560 |
| 2487133 | Chr08 | 7894351 | Potri.008G121300 | GO:0015031 PF03398 | AT1G25420, AT1G34220, AT4G35730 |
| 2691420 | Chr09 | 3535649 | Potri.009G023800 | GO:0046983 PF00010 | - |
| 2725774 | Chr09 | 7211133 | Potri.009G073800 | GO:0030246 GO:0003824 GO:2001070 GO:0003873 GO:0006000 GO:0005524 GO:0006003 PF01591  PF00300 PF00686 | AT1G07110 |
| 2725819 | Chr09 | 7214060 | Potri.009G073800 | GO:0030246 GO:0003824 GO:2001070 GO:0003873 GO:0006000 GO:0005524 GO:0006003 PF01591 PF00300 PF00686 | AT1G07110 |
| 2725846 | Chr09 | 7215386 | Potri.009G073800 | GO:0030246 GO:0003824 GO:2001070 GO:0003873 GO:0006000 GO:0005524 GO:0006003 PF01591 PF00300 PF00686 | AT1G07110 |
| 2918466 | Chr10 | 11272851 | Potri.010G089050 | GO:0048038 GO:0005507 GO:0055114 GO:0009308 GO:0008131 PF01179 PF02727 PF02728 | AT1G31670, AT1G31690, AT1G31710, AT1G62810, AT3G43670, AT4G12270, AT4G12290, AT4G14940 |
| 2919679 | Chr10 | 11493454 | Potri.010G091500 | GO:0003824 GO:0004722 GO:0005515 GO:0006470 PF00498 PF00481 | AT5G19280 |
| 2919720 | Chr10 | 11496619 | Potri.010G091500 | GO:0003824 GO:0004722 GO:0005515 GO:0006470 PF00498 PF00481 | AT5G19280 |
| 2919736 | Chr10 | 11498082 | Potri.010G091500 | GO:0003824 GO:0004722 GO:0005515 GO:0006470 PF00498 PF00481 | AT5G19280 |
| 3073639 | Chr11 | 6083941 | Potri.011G066200 | GO:0048544 PF00954 PF01453 | AT1G16905, AT1G78820, AT1G78830, AT1G78850, AT1G78860 |
| 3104167 | Chr11 | 7884969 | Potri.011G079400 | PF07779 | AT1G29890, AT2G34410, AT3G06550, AT5G46340 |
| 3198153 | Chr11 | 14453981 | Potri.011G119300 | PF04765 | AT1G28240, AT1G53040 |
| 3524391 | Chr13 | 6841205 | Potri.013G078600 | GO:0016773 PF00294 | AT4G27600 |
| 3703543 | Chr14 | 7566434 | Potri.014G096700 | GO:0005975 GO:0008061 PF00704 | AT4G01040 |
| 3719550 | Chr14 | 9178325 | Potri.014G118100 | GO:0008270 GO:0003676 | AT3G62330 |
| 3736636 | Chr14 | 10798417 | Potri.014G141800 | GO:0016787 GO:0004721 GO:0005515 GO:0006470 PF00149 PF08321 PF00515 PF13432 | AT2G42810 |
| 3810850 | Chr14 | 15369575 | Potri.014G182000 | GO:0005975 GO:0004553 PF00332 | AT2G16230, AT2G39640, AT3G46570, AT3G55430, AT4G34480, AT5G24318, AT5G42720 |
| 3931481 | Chr15 | 3269102 | Potri.015G037000 | GO:0050661 GO:0005975 GO:0051287 GO:0016832 GO:0003824 GO:0008270 GO:0055114 GO:0016491 PF03446 PF07005 PF14833  PF14833 | AT1G18270 |
| 4147197 | Chr16 | 3584946 | Potri.016G054800 | GO:0005975  GO:0004650 PF00295 | AT1G23450, AT1G23460, AT1G70500, etc. (17) |
| 4266046 | Chr16 | 11584428 | Potri.016G112600 | PF13439 PF00534 | AT5G01220 |
| 4266112 | Chr16 | 11587224 | Potri.016G112600 | PF13439 PF00534 | AT5G01220 |
| 4266170 | Chr16 | 11589245 | Potri.016G112600 | PF13439 PF00534 | AT5G01220 |
| 4317096 | Chr17 | 1442935 | Potri.017G017000 | PF10294 | AT1G08125, AT5G44170 |
| 4495292 | Chr17 | 15569547 | Potri.017G149900 | GO:0016192 GO:0030008  PF04099 | AT1G51160 |
| 4495312 | Chr17 | 15569708 | Potri.017G149900 | GO:0016192 GO:0030008 PF04099 | AT1G51160 |
| 4495313 | Chr17 | 15569711 | Potri.017G149900 | GO:0016192 GO:0030008 PF04099 | AT1G51160 |
| 4495314 | Chr17 | 15569720 | Potri.017G149900 | GO:0016192 GO:0030008 PF04099 | AT1G51160 |
| 4495315 | Chr17 | 15569767 | Potri.017G149900 | GO:0016192 GO:0030008 PF04099 | AT1G51160 |
| 4495319 | Chr17 | 15569822 | Potri.017G149900 | GO:0016192 GO:0030008 PF04099 | AT1G51160 |
| 4495330 | Chr17 | 15570203 | Potri.017G149900 | GO:0016192 GO:0030008 PF04099 | AT1G51160 |
| 4519045 | Chr18 | 1435873 | Potri.018G018800 | GO:0003860 PF16113 | AT1G06550, AT2G30650, AT2G30660, AT3G60510, AT4G31810, AT5G65940 |
| 4535569 | Chr18 | 2941552 | Potri.018G036500 | GO:0003682 GO:0003676 PF00076 PF01426 | AT3G15605, AT5G11470 |
| 4535609 | Chr18 | 2943109 | Potri.018G036500 | GO:0003682 GO:0003676 PF00076 PF01426 | AT3G15605, AT5G11470 |
| 4535634 | Chr18 | 2944839 | Potri.018G036500 | GO:0003682 GO:0003676 PF00076 PF01426 | AT3G15605, AT5G11470 |
| 4535653 | Chr18 | 2945863 | Potri.018G036500 | GO:0003682 GO:0003676 PF00076 PF01426 | AT3G15605, AT5G11470 |
| 4536144 | Chr18 | 2963586 | Potri.018G036600 | GO:0003887 GO:0006260 GO:0003676 GO:0003677 GO:0005524 PF00270 PF00271 PF00476 | AT4G32700 |
| 4536279 | Chr18 | 2970662 | Potri.018G036600 | GO:0003887 GO:0006260 GO:0006261 GO:0003676 GO:0003677 GO:0005524 PF00270 PF00271 PF00476 | AT4G32700 |
| 4536426 | Chr18 | 2978300 | Potri.018G036600 | GO:0003887 GO:0006260 GO:0006261 GO:0003676 GO:0003677 GO:0005524 PF00270 PF00271 PF00476 | AT4G32700 |
| 4587814 | Chr18 | 6151340 | Potri.018G058000 | GO:0005787 GO:0008233 GO:0016021 GO:0006465 PF04573 | AT3G05230, AT5G27430 |
| 4709516 | Chr19 | 1201140 | Potri.019G010000 | PF00226 | AT2G18465, AT2G42065, AT2G42080, AT3G58020 |
| 4709524 | Chr19 | 1201551 | Potri.019G010000 | PF00226 | AT2G18465, AT2G42065, AT2G42080, AT3G58020 |
| 4824600 | Chr19 | 10223305 | Potri.019G068900 | PF07738 | AT1G22882, AT1G71360, AT4G23950 |
| 4843715 | Chr19 | 11729584 | Potri.019G084900 | GO:0016746 PF02817 PF00364 PF00198 | AT1G34430, AT3G25860 |
| 4845386 | Chr19 | 11818094 | Potri.019G085900 | PF11961 PF05003 | AT1G30755, AT1G34320, AT5G08660 |
| 4855429 | Chr19 | 12838677 | Potri.019G098500 | GO:0043531 GO:0007165 GO:0005515 PF07725 PF00931 PF01582 | AT1G17600, AT1G17610, AT1G17615,  etc. (102) |
| 4855431 | Chr19 | 12838712 | Potri.019G098500 | GO:0043531 GO:0007165 GO:0005515 PF07725 PF00931 PF01582 | AT1G17600, AT1G17610, AT1G17615,  etc. (102) |

**S Table 4.** QTL information within candidate genes that significantly regulate qP.

| SNP | Chr | Position | Gene | Function in *Populus trichocarpa* | Homologous gene in  *Arabidopsis thaliana* |
| --- | --- | --- | --- | --- | --- |
| 167870 | Chr01 | 13870041 | Potri.001G165300 | - | - |
| 371814 | Chr01 | 26610882 | Potri.001G256500 | - | AT2G19220, AT3G11290, AT3G11300, AT3G11310, AT5G05800 |
| 586494 | Chr01 | 40039387 | Potri.001G385200 | GO:0006468 GO:0005524 GO:0005515 GO:0004672 PF13855  PF11721  PF00069 | AT1G07650, AT1G29720, AT1G29730, etc. (14) |
| 651482 | Chr01 | 44547270 | Potri.001G420100 | - | - |
| 768114 | Chr02 | 6881691 | Potri.002G095700 | GO:0006468 GO:0005524 GO:0005515 GO:0004672 PF00560 PF13855 PF00069 | AT1G35710, AT4G08850 |
| 935526 | Chr02 | 20858586 | Potri.002G221500 | PF16312 | AT3G07780, AT5G48160 |
| 999134 | Chr03 | 324750 | Potri.003G003100 | PF05212 | AT1G11170, AT1G61240, AT4G12840, AT4G18530 |
| 999135 | Chr03 | 324795 | Potri.003G003100 | PF05212 | AT1G11170, AT1G61240, AT4G12840, AT4G18530 |
| 999221 | Chr03 | 328922 | Potri.003G003300 | GO:0003824 PF00857 | AT5G23220, AT5G23230 |
| 1275794 | Chr04 | 1815402 | Potri.004G025650 | GO:0006468 GO:0005524 GO:0004672 PF07714 | AT1G11280, AT1G11300, AT1G11303,  etc. (80) |
| 1336811 | Chr04 | 6820836 | Potri.004G082601 | GO:0006606 | - |
| 1514188 | Chr04 | 19865126 | Potri.004G182100 | GO:0004568 GO:0006032 GO:0005975 GO:0008061 GO:0016998 PF00187 | AT1G02360, AT3G12500, AT4G01700 |
| 1514190 | Chr04 | 19865135 | Potri.004G182100 | GO:0004568 GO:0006032 GO:0005975 GO:0008061 GO:0016998 PF00187 | AT1G02360, AT3G12500, AT4G01700 |
| 1591572 | Chr05 | 2231536 | Potri.005G030000 | GO:0005092 GO:0005968 GO:0007264 GO:0018344 GO:0006886 PF00996 | AT3G06540 |
| 1792718 | Chr05 | 17961429 | Potri.005G168750 | - | - |
| 1836011 | Chr05 | 21088527 | Potri.005G193701 | - | AT1G43245 |
| 1885809 | Chr06 | 835322 | Potri.006G012600 | - | - |
| 1979110 | Chr06 | 9145689 | Potri.006G116400 | PF12776 | AT2G19220, AT3G11290, AT3G11300, AT3G11310, AT5G05800 |
| 1979111 | Chr06 | 9145694 | Potri.006G116400 | PF12776 | AT2G19220, AT3G11290, AT3G11300, AT3G11310, AT5G05800 |
| 1991337 | Chr06 | 9964822 | Potri.006G123900 | GO:0016887 GO:0016021 GO:0042626 GO:0005524 GO:0055085 PF00005 PF00664 | AT1G10680, AT1G27940, AT1G28010, AT2G36910, AT3G28345, AT3G28360, AT3G28380, AT3G28390, AT3G28415, AT3G28860, AT4G25960 |
| 2255002 | Chr07 | 2939216 | Potri.007G037500 | PF08268 | AT1G64290, AT1G64295, AT2G05360, AT5G41720 |
| 2255007 | Chr07 | 2939283 | Potri.007G037500 | PF08268 | AT1G64290, AT1G64295, AT2G05360, AT5G41720 |
| 2309065 | Chr07 | 6460490 | Potri.007G060100 | GO:0016409 PF01529 | AT3G51390 |
| 2309103 | Chr07 | 6460710 | Potri.007G060100 | GO:0016409 PF01529 | AT3G51390 |
| 2380395 | Chr07 | 11990973 | Potri.007G093000 | GO:0005515 PF03000 PF00651 | AT5G10250, AT5G64330 |
| 2425585 | Chr07 | 15499717 | Potri.007G145800 | GO:0004649 GO:0005975 PF05028 | AT2G31865, AT2G31870 |
| 2981457 | Chr10 | 18915491 | Potri.010G195600 | GO:0016758 PF00201 | AT1G22340, AT1G22360, AT1G22370,  etc. (28) |
| 3004937 | Chr10 | 22213197 | Potri.010G248900 | GO:0006418 GO:0004831 GO:0004812 GO:0005524 GO:0000166 PF00579 | AT1G28350, AT2G33840 |
| 3103244 | Chr11 | 7859799 | Potri.011G079200 | GO:0008270 GO:0003676 | - |
| 3103249 | Chr11 | 7859821 | Potri.011G079200 | GO:0008270 GO:0003676 | - |
| 3245608 | Chr12 | 309751 | Potri.012G003500 | GO:0006468 GO:0005524 GO:0004672 PF00069 | AT1G34300, AT1G36005, AT1G66910,  etc. (18) |
| 3333075 | Chr12 | 8221270 | Potri.012G064800 | - | - |
| 3333080 | Chr12 | 8221322 | Potri.012G064800 | - | - |
| 3725133 | Chr14 | 9720458 | Potri.014G126500 | PF01190 | AT1G54970, AT2G47530, AT2G47540, AT3G62680, AT4G02270 |
| 3725136 | Chr14 | 9720475 | Potri.014G126500 | PF01190 | AT1G54970, AT2G47530, AT2G47540, AT3G62680, AT4G02270 |
| 3950438 | Chr15 | 4346356 | Potri.015G043600 | GO:0005515 GO:0043531 GO:0007165 PF00931 PF01582 | AT1G17600, AT1G17610, AT1G17615,  etc. (102) |
| 4289987 | Chr16 | 13305342 | Potri.016G130600 | GO:0003924 GO:0005525 PF00071 | AT4G17160, AT4G17170, AT4G35860 |
| 4289988 | Chr16 | 13305344 | Potri.016G130600 | GO:0003924 GO:0005525 PF00071 | AT4G17160, AT4G17170, AT4G35860 |
| 4289989 | Chr16 | 13305347 | Potri.016G130600 | GO:0003924 GO:0005525  PF00071 | AT4G17160, AT4G17170, AT4G35860 |
| 4289990 | Chr16 | 13305351 | Potri.016G130600 | GO:0003924 GO:0005525 PF00071 | AT4G17160, AT4G17170, AT4G35860 |
| 4289997 | Chr16 | 13305397 | Potri.016G130600 | GO:0003924 GO:0005525  PF00071 | AT4G17160, AT4G17170, AT4G35860 |
| 4305871 | Chr17 | 178179 | Potri.017G003400 | GO:0006468 GO:0005524 GO:0004672 PF00069 | - |
| 4324470 | Chr17 | 2509333 | Potri.017G028100 | GO:0005506 GO:0016705 GO:0055114 GO:0020037 PF00067 | - |
| 4324471 | Chr17 | 2509341 | Potri.017G028100 | GO:0005506 GO:0016705 GO:0055114 GO:0020037 PF00067 | - |
| 4474272 | Chr17 | 13537161 | Potri.017G122500 | - | AT5G38060 |
| 4474307 | Chr17 | 13539007 | Potri.017G122500 | - | AT5G38060 |
| 4505047 | Chr18 | 221592 | Potri.018G003200 | GO:0043531 PF00931 PF18052 | AT1G10920, AT1G50180, AT1G53350,  etc. (20) |
| 4505048 | Chr18 | 221600 | Potri.018G003200 | GO:0043531 PF00931 PF18052 | AT1G10920, AT1G50180, AT1G53350,  etc. (22) |
| 4535545 | Chr18 | 2941018 | Potri.018G036500 | GO:0003682 GO:0003676 PF00076 PF01426 | AT3G15605, AT5G11470 |
| 4706861 | Chr19 | 880313 | Potri.019G007400 | GO:0006468 GO:0005524 GO:0004672 PF07714 | - |
| 4713423 | Chr19 | 1759518 | Potri.019G016100 | - | - |
| 4735048 | Chr19 | 3705570 | Potri.019G032600 | GO:0003824 GO:0004743 GO:0000287 GO:0006096 GO:0030955 PF02887 PF00224 | AT3G04050, AT3G25960, AT3G55650, AT3G55810, AT4G26390, AT5G08570, AT5G56350, AT5G63680 |
| 4735051 | Chr19 | 3705632 | Potri.019G032600 | GO:0003824 GO:0004743 GO:0000287 GO:0006096 GO:0030955 PF02887 PF00224 | AT3G04050, AT3G25960, AT3G55650, AT3G55810, AT4G26390, AT5G08570, AT5G56350, AT5G63680 |
| 4748091 | Chr19 | 4493236 | Potri.019G039300 | PF10225 | AT1G28760, AT3G49840, AT5G67610 |

**S Table 5.** QTL information within candidate genes that significantly regulate qN.

| SNP | Chr | Position | Gene | Function in *Populus trichocarpa* | Homologous gene in  *Arabidopsis thaliana* |
| --- | --- | --- | --- | --- | --- |
| 93512 | Chr01 | 8080319 | Potri.001G101300 | GO:0016787 GO:0006289 GO:0006367 GO:0003677 GO:0003678 GO:0005524 PF16203 PF04851 PF13625 | AT5G41360, AT5G41370 |
| 122017 | Chr01 | 10397045 | Potri.001G128400 | GO:0005515 PF08263 PF13855 PF00560 | AT1G45616, AT1G47890, AT1G71390, etc. (34) |
| 158341 | Chr01 | 13172351 | Potri.001G158200 | PF00226 PF11875 | AT2G35720 |
| 158391 | Chr01 | 13175892 | Potri.001G158200 | PF00226 PF11875 | AT2G35720 |
| 158424 | Chr01 | 13177575 | Potri.001G158200 | PF00226 PF11875 | AT2G35720 |
| 369581 | Chr01 | 26469690 | Potri.001G255000 | GO:0005515 PF13181 PF00226 | AT2G41520, AT5G12430 |
| 499451 | Chr01 | 35217152 | Potri.001G346200 | GO:0050660 GO:0055114 GO:0016491 PF07992 | AT3G09940, AT3G27820, AT3G52880, AT5G03630 |
| 766782 | Chr02 | 6677829 | Potri.002G093300 | GO:0003824 GO:0004619 GO:0006096 GO:0016868 PF00300 | AT1G22170, AT1G78050 |
| 786850 | Chr02 | 9009419 | Potri.002G120000 | GO:0006468 GO:0005524 GO:0004672 PF00069 | - |
| 822055 | Chr02 | 12049211 | Potri.002G160100 | GO:0048236 GO:0070192 | AT1G01690 |
| 822061 | Chr02 | 12049473 | Potri.002G160100 | GO:0048236 GO:0070192 | AT1G01690 |
| 822067 | Chr02 | 12049606 | Potri.002G160100 | GO:0048236 GO:0070192 | AT1G01690 |
| 822079 | Chr02 | 12050441 | Potri.002G160100 | GO:0048236 GO:0070192 | AT1G01690 |
| 822096 | Chr02 | 12051041 | Potri.002G160100 | GO:0048236 GO:0070192 | AT1G01690 |
| 822106 | Chr02 | 12051626 | Potri.002G160100 | GO:0048236 GO:0070192 | AT1G01690 |
| 925893 | Chr02 | 19978646 | Potri.002G216700 | PF12776 | AT2G19220, AT3G11290, AT3G11300, etc. (5) |
| 928358 | Chr02 | 20060429 | Potri.002G217200 | - | AT1G21660, AT1G30280, AT1G75100, etc. (7) |
| 928359 | Chr02 | 20060431 | Potri.002G217200 | - | AT1G21660, AT1G30280, AT1G75100, etc. (7) |
| 928360 | Chr02 | 20060439 | Potri.002G217200 | - | AT1G21660, AT1G30280, AT1G75100, etc. (7) |
| 928365 | Chr02 | 20060499 | Potri.002G217200 | - | AT1G21660, AT1G30280, AT1G75100, etc. (7) |
| 928366 | Chr02 | 20060523 | Potri.002G217200 | - | AT1G21660, AT1G30280, AT1G75100, etc. (7) |
| 928370 | Chr02 | 20060541 | Potri.002G217200 | - | AT1G21660, AT1G30280, AT1G75100, etc. (7) |
| 928388 | Chr02 | 20060799 | Potri.002G217200 | - | AT1G21660, AT1G30280, AT1G75100, etc. (7) |
| 928389 | Chr02 | 20060801 | Potri.002G217200 | - | AT1G21660, AT1G30280, AT1G75100, etc. (7) |
| 1031557 | Chr03 | 3242856 | Potri.003G026800 | GO:0016787 GO:0008242 PF07722 | AT1G78660, AT1G78670, AT1G78680 |
| 1121993 | Chr03 | 9060176 | Potri.003G062300 | PF05553 | AT1G15385 |
| 1201407 | Chr03 | 16540377 | Potri.003G151500 | PF08146 PF12397 | AT3G06530 |
| 1500282 | Chr04 | 18746615 | Potri.004G168100 | GO:004698PF00010 | AT3G19860, AT4G36060 |
| 1588137 | Chr05 | 1902689 | Potri.005G025900 | - | - |
| 1588138 | Chr05 | 1902725 | Potri.005G025900 | - | - |
| 1588139 | Chr05 | 1902731 | Potri.005G025900 | - | - |
| 1588140 | Chr05 | 1902746 | Potri.005G025900 | - | - |
| 1588145 | Chr05 | 1902884 | Potri.005G025900 | - | - |
| 1588149 | Chr05 | 1903274 | Potri.005G025900 | - | - |
| 1588150 | Chr05 | 1903286 | Potri.005G025900 | - | - |
| 1588152 | Chr05 | 1903389 | Potri.005G025900 | - | - |
| 1588157 | Chr05 | 1903664 | Potri.005G025900 | - | - |
| 1588161 | Chr05 | 1903819 | Potri.005G025900 | - | - |
| 1607860 | Chr05 | 3789989 | Potri.005G053100 | GO:0016788 PF00657 | -- |
| 1760875 | Chr05 | 15878310 | Potri.005G158200 | - | AT1G64290, AT1G64295, AT2G05360, AT5G41720 |
| 1795511 | Chr05 | 18442383 | Potri.005G170200 | - | - |
| 1795514 | Chr05 | 18442489 | Potri.005G170200 | - | - |
| 1795597 | Chr05 | 18451676 | Potri.005G170300 | GO:0005515 PF13041 PF01535 | AT1G04840, AT1G06140, AT1G06143,  etc. (102) |
| 1821138 | Chr05 | 20013096 | Potri.005G183800 | GO:0008168 GO:0008171  PF00891 | AT1G21100, AT1G21110, AT1G21120,  etc. (16) |
| 1861125 | Chr05 | 23778605 | Potri.005G228500 | GO:0005515 PF01167  PF00646 | AT1G25280, AT1G43640, AT1G47270,  etc. (9) |
| 1956825 | Chr06 | 7476687 | Potri.006G097600 | PF00534 PF13439 | AT5G01220 |
| 2113698 | Chr06 | 19494598 | Potri.006G180900 | GO:0061575 GO:0006289 GO:0045737 GO:0005675 PF06391 | AT4G30820 |
| 2113699 | Chr06 | 19494605 | Potri.006G180900 | GO:0061575 GO:0006289 GO:0045737 GO:0005675 PF06391 | AT4G30820 |
| 2145968 | Chr06 | 21424079 | Potri.006G199000 | PF04576 | AT2G24140, AT3G11850, AT3G54740, etc. (6) |
| 2154949 | Chr06 | 22079339 | Potri.006G205000 | GO:0006355 GO:0003690 PF02536 | AT2G03050, AT2G34620, AT2G36000 |
| 2196220 | Chr06 | 25713007 | Potri.006G251200 | PF03638 | AT3G04850, AT3G22760, AT3G22780, AT4G14770 |
| 2309065 | Chr07 | 6460490 | Potri.007G060100 | GO:0016409 PF01529 | AT3G51390 |
| 2445348 | Chr08 | 1823153 | Potri.008G032901 | PF06813 | AT2G28120, AT2G39210 |
| 2446561 | Chr08 | 1933836 | Potri.008G035000 | PF10167 | AT2G39170 |
| 2471202 | Chr08 | 5794573 | Potri.008G092700 | GO:0016757 PF04577 | AT2G03360, AT2G03370, AT2G41640, AT3G10320, AT3G57380 |
| 2472897 | Chr08 | 5987614 | Potri.008G095700 | PF04884 | AT1G13770 |
| 2499492 | Chr08 | 9325127 | Potri.008G139700 | GO:0008017 GO:0000226 PF03999 | AT1G14690, AT1G27920, AT2G01910, etc. (9) |
| 2638523 | Chr08 | 19017918 | Potri.008G223600 | PF14111 | AT2G01050 |
| 2765789 | Chr09 | 12162695 | Potri.009G156400 | GO:0006777 GO:0032324 PF00994 PF03454 PF03453 | AT5G20990 |
| 2776601 | Chr10 | 183047 | Potri.010G001600 | GO:0006468 GO:0005524 GO:0004672 PF00069 | AT1G01540, AT1G09440, AT1G56720, etc. (11) |
| 2776610 | Chr10 | 183454 | Potri.010G001600 | GO:0006468 GO:0005524 GO:0004672 PF00069 | AT1G01540, AT1G09440, AT1G56720, etc. (11) |
| 2776614 | Chr10 | 183878 | Potri.010G001600 | GO:0006468 GO:0005524 GO:0004672 PF00069 | AT1G01540, AT1G09440, AT1G56720, etc. (11) |
| 2776615 | Chr10 | 183895 | Potri.010G001600 | GO:0006468  GO:0005524 GO:0004672 PF00069 | AT1G01540, AT1G09440, AT1G56720, etc. (11) |
| 2776630 | Chr10 | 185067 | Potri.010G001600 | GO:0006468  GO:0005524 GO:0004672 PF00069 | AT1G01540, AT1G09440, AT1G56720, etc. (11) |
| 2890607 | Chr10 | 8643582 | Potri.010G056900 | GO:0006468 GO:0005524 GO:0004672 PF00069 | AT1G67580, AT5G63370 |
| 3025904 | Chr11 | 1647709 | Potri.011G019200 | GO:0005515 PF12796 PF13962 | AT3G18670, AT3G54065, AT5G04680, etc. (8) |
| 3025915 | Chr11 | 1647773 | Potri.011G019200 | GO:0005515 PF12796 PF13962 | AT3G18670, AT3G54065, AT5G04680, etc. (8) |
| 3035166 | Chr11 | 2584190 | Potri.011G030900 | GO:0016021 PF01925 | AT1G11540, AT1G61740, AT4G21250, AT4G21260 |
| 3043690 | Chr11 | 3523870 | Potri.011G041500 | PF03109 | AT1G11390, AT1G61640 |
| 3043693 | Chr11 | 3524461 | Potri.011G041500 | PF03109 | AT1G11390, AT1G61640 |
| 3043713 | Chr11 | 3526390 | Potri.011G041500 | PF03109 | AT1G11390, AT1G61640 |
| 3278883 | Chr12 | 4069813 | Potri.012G044701 | GO:0004674 GO:0006468 GO:0005524 GO:0004672 PF00069 | AT1G45160, AT1G48490, AT3G17850, AT5G62310 |
| 3278889 | Chr12 | 4070676 | Potri.012G044701 | GO:0004674 GO:0006468 GO:0005524 GO:0004672 PF00069 | AT1G45160, AT1G48490, AT3G17850, AT5G62310 |
| 3278976 | Chr12 | 4074894 | Potri.012G044701 | GO:0004674 GO:0006468 GO:0005524 GO:0004672 PF00069 | AT1G45160, AT1G48490, AT3G17850, AT5G62310 |
| 3278978 | Chr12 | 4075160 | Potri.012G044701 | GO:0004674 GO:0006468 GO:0005524 GO:0004672 PF00069 | AT1G45160, AT1G48490, AT3G17850, AT5G62310 |
| 3279029 | Chr12 | 4077861 | Potri.012G044701 | GO:0004674 GO:0006468 GO:0005524 GO:0004672  PF00069 | AT1G45160, AT1G48490, AT3G17850, AT5G62310 |
| 3357068 | Chr12 | 9423934 | Potri.012G070300 | GO:0098656 GO:0005741 GO:0055085 GO:0008308 PF01459 | - |
| 3503858 | Chr13 | 5450666 | Potri.013G067700 | GO:0016485 GO:0016021 GO:0004190 PF01080 | AT1G08700, AT2G29900 |
| 3510072 | Chr13 | 5891536 | Potri.013G070500 | GO:0003700 GO:0006355 GO:0006352 PF04545 PF04539 PF04542 | AT1G08540 |
| 3700282 | Chr14 | 7229040 | Potri.014G092500 | PF00534 | AT4G01210, AT5G04480 |
| 3756903 | Chr14 | 12187570 | Potri.014G156300 | GO:0006511 GO:0005515 PF03152 | AT4G15420 |
| 3879982 | Chr14 | 18195708 | Potri.014G194300 | GO:0006468 GO:0005524 GO:0004672 PF07714 | AT2G20300, AT5G56890 |
| 4139921 | Chr16 | 2890120 | Potri.016G045800 | GO:0003887 GO:0006260 GO:0003677 PF18018 PF04042 | AT2G42120 |
| 4143268 | Chr16 | 3210391 | Potri.016G050400 | GO:0003924 GO:0005525 PF00071 | AT1G01200, AT1G05810, AT1G06400,  etc. (25) |
| 4157232 | Chr16 | 4403998 | Potri.016G062600 | GO:0006396 GO:0005634 GO:0003676 GO:0005515 GO:0006397 PF05843 PF00575 | AT3G11964 |
| 4594627 | Chr18 | 7111729 | Potri.018G060900 | - | - |
| 4675667 | Chr18 | 13805045 | Potri.018G112200 | PF03556 | AT1G15860 |
| 4675672 | Chr18 | 13805523 | Potri.018G112200 | PF03556 | AT1G15860 |
| 4692569 | Chr18 | 16374905 | Potri.018G144100 | - | - |
| 4848312 | Chr19 | 12114027 | Potri.019G089500 | - | - |
